# Synthesis and Characterization of Molten Salt Nanofluids for Thermal Energy Storage Application in Concentrated Solar Power Plants—Mechanistic Understanding of Specific Heat Capacity Enhancement

**DOI:** 10.3390/nano10112266

**Published:** 2020-11-16

**Authors:** Binjian Ma, Donghyun Shin, Debjyoti Banerjee

**Affiliations:** 1 School of Mechanical Engineering and Automation, Harbin Institute of Technology (Shenzhen), Shenzhen 518055, China; 2 School of Engineering & Technology, Central Michigan University, Mount Pleasant, MI 48859, USA; shin1d@cmich.edu; 3 Department of Mechanical Engineering, Department of Petroleum Engineering, Mary Kay O’Connor Process Safety Center, Texas A&M University, College Station, TX 77843, USA; dbanerjee@tamu.edu

**Keywords:** molten salt nanofluid, specific heat capacity, one-step synthesis, T-history method, secondary nanostructure

## Abstract

Molten salts mixed with nanoparticles have been shown as a promising candidate as the thermal energy storage (TES) material in concentrated solar power (CSP) plants. However, the conventional method used to prepare molten salt nanofluid suffers from a high material cost, intensive energy use, and laborious process. In this study, solar salt-Al_2_O_3_ nanofluids at three different concentrations are prepared by a one-step method in which the oxide nanoparticles are generated in the salt melt directly from precursors. The morphologies of the obtained nanomaterials are examined under scanning electron microscopy and the specific heat capacities are measured using the temperature history (T-history) method. A non-linear enhancement in the specific heat capacity of molten salt nanofluid is observed from the thermal characterization at a nanoparticle mass concentration of 0.5%, 1.0%, and 1.5%. In particular, a maximum enhancement of 38.7% in specific heat is found for the nanofluid sample prepared with a target nanoparticle mass fraction of 1.0%. Such an enhancement trend is attributed to the formation of secondary nanostructure between the alumina nanoparticles in the molten salt matrix following a locally-dispersed-parcel pattern. These findings provide new insights to understanding the enhanced energy storage capacity of molten salt nanofluids.

## 1. Introduction

With increasing concern about environmental quality and sustainable development, renewable sources of energy such as wind, solar and hydropower are starting to play a significant role in the modern energy supply. Among all sources of renewable energy, solar energy is considered to be the most promising and suitable alternative for supplementing current energy consumption profiles due to the potential for almost zero pollution, and cheap cost of deployment and operation [1]. The theoretical limit for solar power on the surface of the Earth is 89,300 TW [2] which suggests that there is more energy received by the Earth in one and a half hours than the world energy consumption in a year, say in 2013 (i.e., 108,170 TW·h equivalent) [3]. Concentrated solar power (CSP) is a widely used technique for converting solar radiation into electricity. Such a technique has also been successfully commercialized in many different countries. CSP plants use mirrors and lenses to concentrate and focus sunlight onto a thermal receiver. The receiver absorbs and converts sunlight into heat. The heat is then transported to a steam generator or engine where it is converted into electricity. Since the performance of solar energy is greatly affected by the climate condition and there is usually a significant difference in peak time between the power generation period and power demand for electricity, thermal energy storage (TES) has become an important and indispensable part of a CSP plant that can be used to shift off-peak energy need and buffers during transient weather conditions. There are several candidate materials to be used as potential heat transfer fluid (HTF) and TES medium. Among the commonly-used materials, thermal oils have a relatively low density, low heat capacity and low maximum operating temperature of 300 °C which makes power generation quite expensive [4]. Ionic liquid has good thermo-physical properties, excellent chemical stability and little vapor pressure [5]. However, as a result of their high cost, the usage of ionic liquid is still limited. Molten salts, with the high density, low vapor pressure, high operating temperature, low chemical reactivity and moderate cost, has been used as the ideal media for TES applications [6].

While molten salts have shown promising properties for high temperature application in a TES system, most of these salts or their eutectics have a specific heat capacity of less than 2 J/g·K and a density less than 2000 kg/m^3^ [7]. As a consequence, the energy storage density of a molten salt system is less than 1000 MJ/m^3^ for a typical operating temperature between 300 °C and 550 °C. In comparison, the energy storage capacity of a thermochemical energy storage system involving redox cycles of metal oxide can easily exceed 3000 MJ/m^3^ [8]. Due to the low energy density of molten salt, huge storage tanks are often required in CSP plants to provide adequate TES capacity which incurs significant additional operation & maintenance (O&M) cost. According to the report released by the National Renewable Energy Laboratory in January 2019 [9], the 2030 target for CSP baseload plants with a minimum of 12 h of energy storage is $0.05/kW·h which marks more than 60% reduction from the current cost level at $0.182/kW·h [10]. Such a stringent requirement urges the development of advanced energy storage material for TES system at a reasonable production cost.

The progress in nanofabrication and nanofluid technology provides pathways to thermophysical property enhancement without replacing the original material completely. Nanofluids are stable colloidal mixtures of nanoparticles (1~100 nm) with liquid solvent. Back in 1993, the pioneer study by Masuda et al. [11] had already shown that dispersing a small percentage of nanoparticles in solvent can bring anomalous improvement to its thermal conductivity. This work was further extended by Choi and Eastman [12] in 1995 who first used the term “nanofluids” to describe heat transfer fluids containing suspending metallic nanoparticles. Their study demonstrated that the high thermal conductivity of nanofluids can potentially improve the heat transfer rate in a heat exchanger without an increase in the pumping power. Subsequently, a plethora of combinations of nanoparticles and liquids have been studied for enhancing transport phenomena and thermophysical properties. Most often, inclusion of nanoparticles in the base solvent yields improved thermal conductivity [13,14,15,16] but at the cost of greater viscosity [17,18,19]. However, the effect of nanoparticles on the specific heat capacity is more intriguing. For aqueous solvents, the specific heat capacity has been reported to decrease with an increasing concentration of nanoparticles [20,21,22,23]:

(1) $$c_{p,nf}=\varphi_{np}c_{p,np}+\varphi_{f}c_{p,f}$$

where φ is the mass fraction, *c_p_* is the specific heat capacity value, and the subscripts *nf*, *np* and *f* stand for nanofluid, nanoparticle and base fluid. On the other hand, an increasing number of studies have reported a radical enhancement in the specific heat capacity of non-aqueous solvent and in particular molten salt [24,25,26,27,28,29,30]. For example, the early study by Shin and Banerjee reported an enhancement up to 24% in the specific heat capacity of molten salt eutectic containing 1% SiO_2_ nanoparticles [31,32]. The latest study by Baha El Far et al. has also shown a 19% improvement in the specific heat capacity of binary carbonate salt mixture by dispersing SiO_2_ nanoparticles [33].

**Figure 1:**
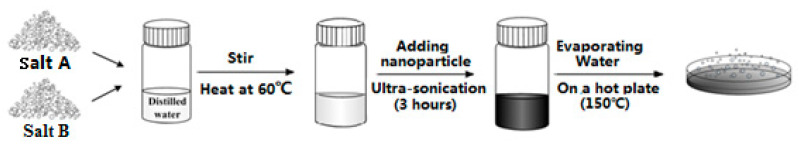
Schematic diagram of two-step synthesis procedure used in past literature studies.

Although adding nanoparticles offers a straightforward method to improve the energy storage capacity of molten salt in the TES system, procuring nanoparticles from vendors and dispersing them in molten salt is not a favorable solution in engineering applications partially due to the high cost of commercially available nanoparticle (~$1000/kg) and partially due to the intense energy use required to dehydrate the molten salt solution during the dispersion process. In particular, the use of water to dissolve the salt can potentially intensify the agglomeration of nanoparticles and introduce impurities to the molten salt system. Figure 1 shows a typical two-step nanofluid synthesis protocol which involves mixing of nanoparticles in the salt solution followed by 2~3 h’ ultra-sonication and evaporation.

In recent years, different synthesis approaches have been developed to overcome the aforementioned issues associated with the traditional wet dissolution method. For example, Grosu and Nithiyanantham et al. [34,35] prepared molten carbonate and nitrate salt-based nanofluid using a dynamic drying mixing approach. In their studies, the eutectic salt mixture was first prepared from the individual salt components through mixing and melting at high temperature. Subsequently, the cooled eutectic salt was ground to find powders and mixed with the nanoparticles in the dry form. Finally, the solid mixture was subjected to a high-energy physical milling process to yield the nanofluid sample. Navarrete et al. prepared molten salt nanofluid using a wet milling method in which the solar salt and nanoparticles were milled in a ball mill with acetone [36]. A couple of other studies have achieved direct mixing of nanoparticles in liquified molten salt at high temperature (i.e., above the melting point of the salt eutectic). For example, Chieruzzi et al. [37] prepared molten salt nanofluids by mixing SiO_2_ and Al_2_O_3_ nanoparticles with NaNO_3_-KNO_3_ solar salt at 300 °C in a micro-conical twin screw compounder. Chen et al. [38] dispersed SiO_2_ nanoparticles in liquified quaternary nitrate molten salt at 400 °C using a magnetic stirrer. While these direct dispersion approaches eliminate the use of water in the synthesis process, prefabricated nanoparticles are still required to prepare the nanofluid samples. As an alternative, a number of studies have proposed the concept of synthesizing nanoparticles in-situ in the molten salt from cost-effective precursors [39,40,41,42]. In other words, the synthesis and dispersion of nanoparticles occur simultaneously in the solvent phase. As a consequence, the propensity of nanoparticle agglomeration is minimized when the nanofluid is prepared following this one-step method [43]. However, it is more difficult to control the morphology of the particles precisely as small variations in the designed synthesis conditions (temperature, time, feeding rate, etc.) can drastically alter the properties of the synthesized nanofluids due to variation in nanoparticle size distribution and stability. Therefore, the optimal synthesis condition for preparing molten nanofluid with enhanced specific heat capacity remains unclear. In particular, the optimal target concentration of nanoparticles to be synthesized in-situ in molten salt remains to be determined. The choice of the proper nanoparticle concentration requires a mechanistic understanding on the enhanced thermophysical property of molten salt nanofluids. In this study, solar salt (NaNO_3_-KNO_3_ eutectic)-based nanofluids containing Al_2_O_3_ nanoparticles at three different concentrations are prepared by a one-step method in which the oxide nanoparticles are generated in the salt melt directly from precursors. The morphological structures of the nanomaterials obtained are examined under scanning electron microscopy and the specific heat capacities are measured using the temperature history (T-history) method. Finally, a hypothesized model is developed to rationalize the enhanced specific heat capacity of molten salt nanofluids.

## 2. Materials and Methods

### 2.1. Sample Preparation

In this study, the binary mixture of 60-wt.% NaNO_3_ and 40-wt.% KNO_3_ (solar salt) was used as the base fluid for the nanofluid synthesis. The one-step method combines the nanoparticle generation and dispersion into one single step in which nanoparticles are synthesized in the base fluid in-situ. This can be achieved by either a physical approach or a chemical approach. One typical physical approach is vapor condensation in which metal vapor is directly condensed to form nanoparticles inside the base fluid by contacting the flowing vapor (at low pressures) within the liquid [12]. Other innovative physical methods include submerged arc spray synthesis [44] and laser ablation [45]. These methods produce well-dispersed nanofluids but require a complex set up which makes it impossible for scale-up and large-scale deployment in industrial applications. By contrast, the wet chemical approach offers a more convenient synthesis method in which chosen additives are mixed with the base fluid and subsequently stimulated to yield nanoparticles. Some typical wet chemistry-based synthesis methods include the chemical reduction method, the precipitation (ion exchange) method, the sol-gel (hydrolysis) method, and the emulsion-polymerization method [43]. These methods have also been proven to produce well-dispersed nanofluids with controllable particle size and shape. However, the direct precipitation method usually requires a different base fluid from that of the target nanofluid (i.e., molten salt in this case). Also, the introduction of additives risks the inadvertent contamination by impurities. In this study, metal oxide nanoparticles were synthesized in high temperature molten salt in-situ by thermal decomposition of precursors (e.g., unstable salts) mixed a priori with the salt powders. Solar salts are shown to be stable in liquid form up to 600 °C [46] and the precursor are expected to decompose and yield corresponding nanoparticles that are self-dispersed in the motlen salt system. Prior to the test, the decomposition temperature and mass loss of more than 10 candidate precursors were explored initially by thermogravimetric (TGA) analysis in a flux of nitrogen at 100 mL/min with a scanning rate of 5 °C/min (SDT Q600, TA Instrument, New Castle, DE, USA, see Supplementary Material S1 for the detailed information). Finally, aluminum nitrate nonahydrate was selected as the optimum test precursor due to its low decomposition temperature and sharing the same anion with the molten salt solvent. In other words, no exterior contaminations (e.g., chloride ions) are explicitly introduced into the molten nitrate salt system after the precursor decomposes.

The synthesis started with mixing all salt components with the precursors in solid powder form in the container. The mixture was stirred for around 1 min and baked in furnace at 550 °C directly for 10 h to ensure complete reaction. As shown by the TGA test results, a complete decompostion of Al(NO_3_)_3_·9H_2_O into Al_2_O_3_ is attained at 400 °C where the final mass percentage left is 13.6%. Therefore, heating the mixture at 550 °C can ensure a full decomposition of the precursor. Three different target mass fraction of alumina nanoparticles including 0.5%, 1% and 1.5% were explored in this study. The quantity of the precursor and salt powder used in the synthesis are listed in Table 1 in which the mass of the nanoparticle precursor was determined from the stoichiometric values for the target mass fraction of Al_2_O_3_ nanoparticles based on the assumption of complete thermal degradation of the precursors. All chemicals were purchased directly from Sigma-Aldrich Inc. (St. Louis, MO, USA) with a reagent grade ~99% purity and used directly without further purification.

### 2.2. Material Characterization

Scanning electron microscopy (SEM) coupled with energy-dispersive X-ray spectroscopy (EDS) techniques were used for materials characterization of the nanofluids samples. For SEM, two scanning electron microscopes including QUANTA 600 FE-SEM (FEI Company, Hillsboro, OR, USA) and JSM-7500F (JEOL Ltd., Akishima, Tokyo, Japan) were used to analyze the microstructure of the samples. The comparison of the images obtained from SEM for pure salt samples and additive-doped samples enable the visualization of the dispersion and morphological characteristics of nanoparticles generated in these samples. The following procedures were followed for preparing the sample for SEM characterization:

⬝ Heat the container with sample on hot plate at 400 °C;
⬝ Once the sample melt, remove them from hot plate and keep stirring/scratching the sample using the spatula to prevent them agglomerating in the container;
⬝ Load ~20 mg of sample in the aluminum pan;
⬝ Heat the pan on hot plate at 400 °C for few seconds until the sample melt, then quickly seal the pan with lid;
⬝ Put the newly-prepared sample pan in a furnace and heat at 550 °C for half hour;
⬝ Remove the pan from the furnace. Wait it cool down and take to the SEM room;
⬝ When examining sample in SEM facility, remove the lid and place the sample pan in SEM chamber quickly to avoid absorption of moisture from the ambient.

EDS was performed to identify the elemental composition for different regions of the pure molten salts and molten salt nanofluid samples. To be more specific, a regional EDS scan is employed to obtain elemental mapping. In order to highlight the nano-cluster areas, point EDS analysis is employed to differentiate between the chemical composition in the nano-cluster areas and the bulk salt areas.

### 2.3. Specific Heat Capacity Measurement

**Figure 2:**
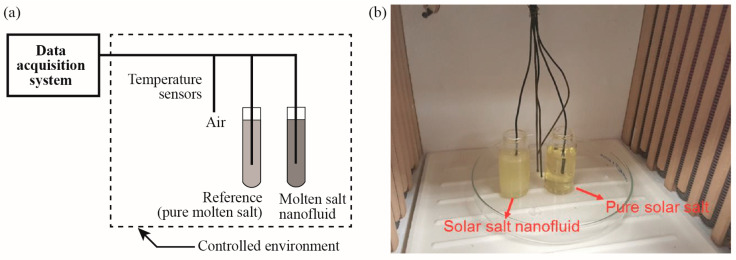
(**a**) Schematic of the T-history system; (**b**) experimental apparatus for T-history measurements.

The specific heat capacity of the different samples were measured using a temperature-history (T-history) method [47]. In the T-history method, the test sample and a reference sample were heated under the same environmental conditions and the specific heat capacity ratio between these two materials can then be calculated based on the difference in their temperature scanning rates. Such a method was originally developed for characterizing the thermophysical properties of phase change materials due to its capability of measuring the specific heat capacity and latent heat of a large quantity sample (>10 g). By contrast, the conventional differential scanning calorimetry (DSC) method only allows testing of samples at milligram scale which is less representative of the bulk energy storage capacity of the material in a TES facility. Figure 2 shows the actual setup of the T-history test in which two vials were filled with pure solar salt and solar salt nanofluid, respectively. The masses of both samples were controlled to be 30 g using a Sartorius ELT-130 microbalance (Sartorius AG, Göttingen, Germany) and the temperature of each sample was measured using a thermocouple (10 KMQXL-062U-12, standard limit of error = ±2.2 °C or 0.75%, Omega Engineering, Norwalk, CT, USA) inserted in the middle of sample. Meanwhile, the environmental temperature in the furnace was measured using three thermocouples installed at different locations. Prior to the actual test, the testing and reference salt sample were preheated at 275 °C for 1 h to ensure a steady state condition. Afterwards, the furnace temperature was set to increase from 275 °C to 650 °C with a heating rate of 20 °C/m. At the same time, the temperature changes of both samples were recorded at a sampling rate of 5 Hz starting from the initial equilibration temperature (~275 °C) to an upper temperature limit of 550 °C. As soon as the temperature of the test sample approaches 550 °C, the temperature recording is stopped, and the furnace is switched off and open to the ambient which allows the test samples to cool down. After the temperature of the molten salt drops between 300 °C, the furnace is then re-set to 275 °C and the testing cycle is repeated following the same sequence.

As discussed earlier, the specific heat capacity ratio between molten salt nanofluids and pure molten salt can be calculated by comparing the temperature history curve (*T* vs. *t*) of both nanofluid sample and the reference salt sample. However, it is important to ensure that the temperature distribution is uniform in the salt sample during the test. In our setup, a 1.0-inch diameter vial with a nominal height of 1.25 inch was used to contain the salt samples. Therefore, the characteristic length (volume/area) of the container is calculated to 0.178 inch. As shown in our early study [16], the thermal conductivity of the pure solar salt was measured to range from 0.43 W/(m·K) to 0.56 W/(m·K) as the temperature increases from 300 °C to 500 °C. Now, assuming a thermal conductivity of 0.5 W/(m·K) and a natural convection heat transfer coefficient of 5 W/(m^2^·K), the Biot number of the pure molten salt sample during the T-history test is calculated to be 0.045. Since molten salt nanofluid exhibits a higher thermal conductivity, the Biot number of the molten salt nanofluid sample will be even smaller than 0.045. Thus, both the pure salt and nanofluid sample satisfy the lumped capacitance assumption and we can assume that the temperature of both samples remains spatially uniform in the container during the test. During the T-history test, the test tubes are heated by both natural convection from the air and conduction from the bottom plate. The thermal resistance associated with natural convection on the side wall of the test tube is given by:

(2) $$R_{side}=\frac{1}{h_{air}A_{side}}$$

where, $h_{air}$ is the natural convection heat transfer coefficient and $A_{side}$ is the surface area of the side wall of the test tube. The conduction heat transfer through the bottom plate can be treated as an annual fin problem and the associated thermal resistance is given by:

(3) $$R_{bottom}=\frac{1}{h_{air}A_{f}\eta_{f}}$$

where $A_{f}$ is the surface area of the glass disk exposed to air and $\eta_{f}$ is the fin efficiency of the circular disk. Based on the geometric parameters of the experimental setup used in this study, the fin efficiency of the glass disk is estimated to be 25% (see Supplementary Material S2 for the detailed calculation). Therefore, during the heating process, the total thermal resistance of both test samples is given by:

(4) $$R_{tot}=\frac{1}{\frac{1}{R_{side}}+\frac{1}{R_{bottom}}}=\frac{1}{h_{air}\left(A_{side}+A_{f}/4\right)}$$

In this way, the temperature scanning rate of both samples could be explicitly expressed by:

(5) $$T_{s}^{\prime }=\frac{dT_{s}}{dt}=\frac{h_{air}\left(A_{side}+A_{f}/4\right)\left(T_{air}-T_{s}\right)}{m_{s}c_{p,s}}$$

where, *T_air_* is the instantaneous temperature of furnace air obtained by taking the average measurements from the three thermocouples, *T_s_* is the instantaneous temperature of the testing sample, *m_s_* is the mass of the testing sample, and *c_p,s_* is the specific heat capacity of the sample. In our test, $\left(A_{side}+A_{f}/4\right)$ remains the same for both the pure molten salt sample and the nanofluid sample.

Considering that the variation of natural convective heat transfer coefficient between two samples is negligible at the same sample temperature, the ratio of specific heat capacity of two samples at any temperature can be expressed by:

(6) $$\frac{c_{p,nano}}{c_{p,ref}}=\frac{m_{ref}}{m_{nano}}\cdot \frac{{\left(\frac{dT_{s}}{dt}\right)}_{ref}}{{\left(\frac{dT_{s}}{dt}\right)}_{nano}}\cdot \frac{{\left(T_{air}-T_{s}\right)}_{nano}}{{\left(T_{air}-T_{s}\right)}_{ref}}\;\;\;\;\;\;\;\;\;\;\mathrm{at}\;\mathrm{any}\;\mathrm{fixed}\;\;T_{s}$$

where, *m_ref_* and *m_nano_* are the mass of the reference sample (pure molten salt) and nanofluid sample, respectively. Prior to the experimental test, the T-history method was first validated by measuring the specific heat capacity of propylene glycol and isopropyl alcohol. Both results are in excellent agreement with the literature measurements (see Supplementary Material S3 for detailed information). While molten salts may evaporate at elevated temperature, the evaporative mass losses of the pure solar salt and solar salt nanofluid were measured to be less than 1.0% per hour (see Supplementary Material S4 for the detailed analysis) at a constant temperature of 550 °C. Considering that the T-history test generally lasted for less than 0.3 h and the salt temperature was slowly increased from 275 °C to 550 °C, *m_ref_* and *m_nano_* can therefore be assumed to be constant during the test. During data analysis, the time derivative of the temperature is obtained by fitting the temperature history curve with an 8th order polynomial function.

## 3. Results

### 3.1. Thermophysical Properties

**Figure 3:**
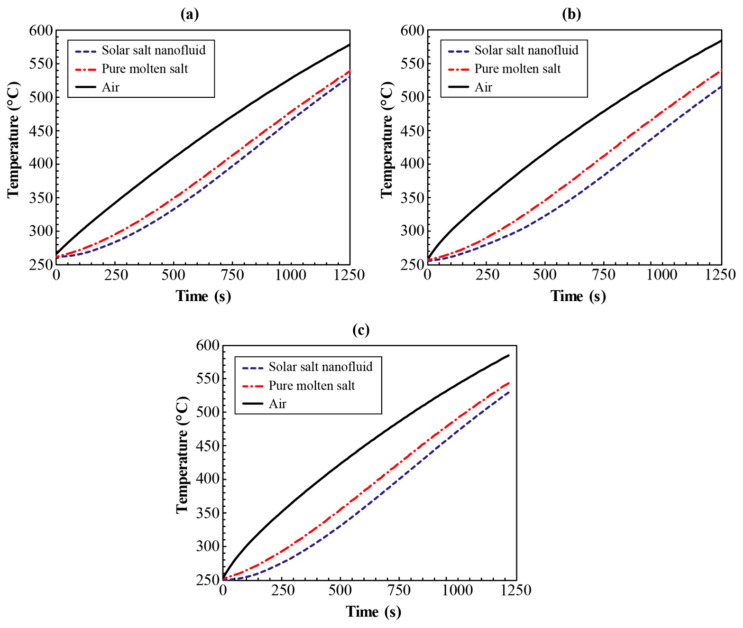
Temperature response recorded during T-history experiments for temperature of air, reference sample (pure solar salt) and test sample (pure solar salt nanofluid with alumina nanoparticles generated from thermal decomposition of aluminum nitrate nonahydrate additives). The mass fraction of additive (alumina nitrate) used and the target mass fraction alumina nanoparticles, are: (**a**) 3.5% and 0.5%, respectively; (**b**) 6.9% and 1%, respectively; and (**c**) 10.1% and 1.5%, respectively.

Figure 3 shows the thermocouple response recorded during T-history experiments for temperature of air, reference sample (pure solar salt) and test sample (pure solar salt nanofluid with alumina nanoparticles generated from thermal decomposition of aluminum nitrate nonahydrate additives). The samples were synthesized for three different concentrations of the alumina nitrate additive (3.5%, 6.9% and 10.1%; with target mass fraction of the alumina nanoparticles of 0.5%, 1% and 1.5%). A total of 10 repeated tests were conducted for each nanoparticle concentration (see Supplementary Material S5 for the complete temperature response of the molten salt nanofluids samples recorded over continuous 10 test cycles). As shown in the figure, there is a substantial time lag in the temperature rise of the molten salt nanofluid sample compared to that of the pure molten salt. The faster temperature response of the solar salt sample provides direct evidence of the enhanced thermal energy storage capacity of the molten salt nanofluid sample. At any given instant during the heating cycle, it is evident that the solar salt sample was at a higher temperature than the nanofluid sample which represents a smaller temperature gradient formed between the pure salt sample and the ambient air. Therefore, less heat is being transported from the ambient air to the pure molten salt sample. Meanwhile, visual inspection suggests that the temperature curves of both the pure salt and nanofluid sample share similar magnitude of slope during the intermediate and later stage of the heating process. These features indicate that the molten salt nanofluid exhibits a higher specific heat capacity than the pure solar salt sample considering that the mass of the molten salt used in the test is almost identical to that of the solar salt nanofluid. Furthermore, as shown by the comparison between the three subplots in Figure 3, the mismatch between the temperature curves between the pure solar salt samples and the nanofluid sample is minimized at a nanoparticle concentration of 0.5%. As suggested by this characteristic, the nanofluid samples synthesized with a target nanoparticle mass fraction of 0.5% exhibit the lowest specific heat capacity among all three concentrations explored in this study. By contrast, the highest specific heat capacity is found for the nanofluid sample synthesized with a target nanoparticle mass fraction of 1%.

**Figure 4:**
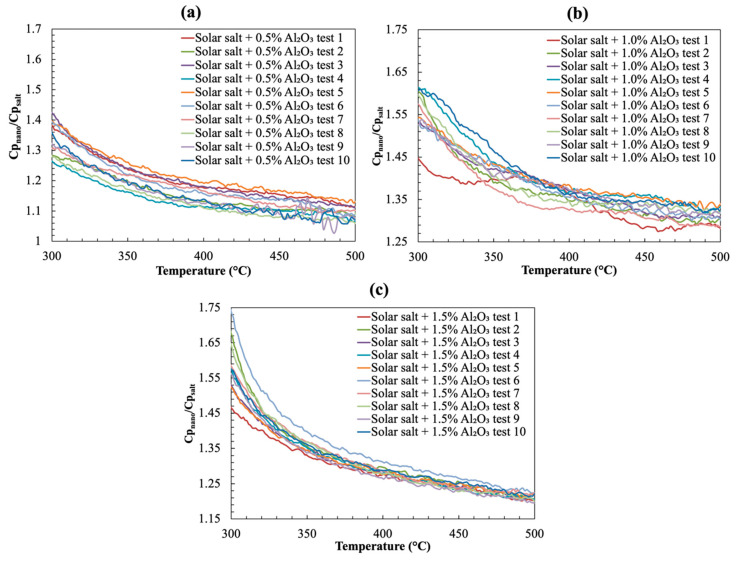
The specific heat capacity ratio between the nanofluid samples and the pure solar salt sample. The mass fraction of additive (alumina nitrate) used and the target mass fraction alumina nanoparticles, are: (**a**) 3.5% and 0.5%; (**b**) 6.9% and 1%; and (**c**) 10.1% and 1.5%, respectively.

The specific heat capacity enhancement of the molten salt nanofluid samples synthesized at three different nanoparticle concentrations can be calculated using Equation (6) based on the information shown in Figure 3. The corresponding results are summarized in Figure 4. As shown in the plots, the level of enhancement is reduced slightly with increasing temperature but remains relatively constant when approaching the maximum testing temperature. To analyze the results in detail, the average specific heat capacity ratio ($\bar{SR}$) between the nanofluid and pure molten salt sample and the corresponding standard deviations *(*$\sigma_{SR}$) for each test cycle were calculated by:

(7) $$\bar{SR}=\frac{\sum_{i=0}^{n}SR_{i}}{n}$$

(8) $$\;\sigma_{SR}=\frac{\sum_{i=0}^{n}SR_{i}}{n}\;$$

where *n* is the total number of temperature data points recorded between 300 °C and 500 °C in each test cycle. The grand average specific heat ratio (Grand Avg) and the grand standard deviation (Grand STD) for the entire test results were calculated using the same set of equations but incorporating all the data points collected over the 10 repeated test cycles (e.g., n becomes the total number of temperature data points recorded between 300 °C and 500 °C in 10 repeated test cycles). The results are shown in Table 2. While certain level of data scattering was observed between the repeated test cycles, the grand standard deviation was found to be in the same level of the stand deviation calculated from each test cycle which suggests a good repeatability of the T-history test. In addition, the tests of the three different molten salt nanofluid samples were affected by a consistent systematic uncertainty. Using one standard deviation, the specific heat capacity of nanofluid samples with 0.5%, 1.0% and 1.5% target mass concentration of Al_2_O_3_ nanoparticles were enhanced by (17.4 ± 7.5)%, (38.8 ± 7.5)%, and (31.8 ± 9.7)%, respectively, with a 68% confidence interval. It is evident that specific heat capacity enhancement was most significant when the nanoparticle concentration is 1.0%. Assuming a continuous change of specific heat capacity with increasing nanoparticle concentration, it is inferred that the optimal concentration for maximizing the specific heat capacity enhancement for the solar salt nanofluids is in excess of 0.5% and less than or equal to 1%. To further validate the results obtained from the T-history test, the specific heat capacities of the molten salt nanofluid sampling containing 1.0% nanoparticles were also tested by modulated differential scanning calorimetry (MDSC) at another facility independently. The results demonstrated that the T-history experiments are consistent with the MDSC experiments with a relative error less than 1% (see Supplementary Material S6 for detailed MDSC measurements).

### 3.2. Material Morphology

**Figure 5:**
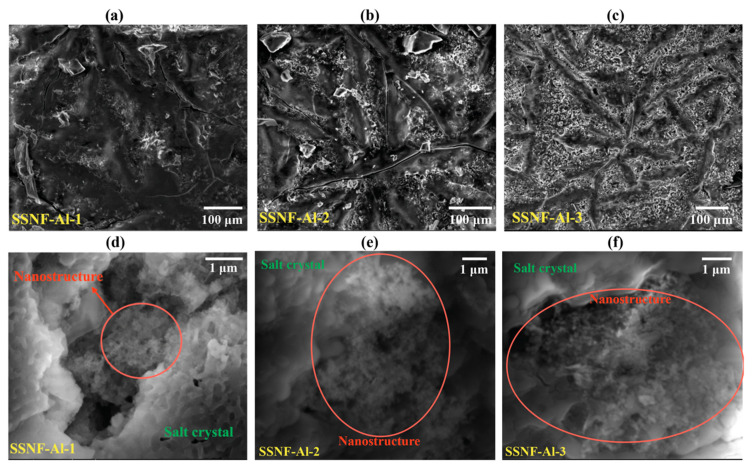
Scanning electron microscopy (SEM) image of the molten salt nanofluid samples. For samples shown in (**a**,**d**), the mass fraction of additive (alumina nitrate) used and the target mass fraction alumina nanoparticles are 3.5% and 0.5%, respectively; for samples shown in (**b**,**e**), the mass fraction of additive used and the target mass fraction alumina nanoparticles are 6.9% and 1.0%, respectively; for samples shown in (**c**,**f**), the mass fraction of additive used and the target mass fraction alumina nanoparticles are 10.1% and 1.5%, respectively.

Figure 5 shows the SEM images of solar salt nanofluid samples at varying levels of magnification. At low magnification (~400×), ridge-shaped microstructures are observed in these images and the number of these ridge microstructures increases drastically with increasing value of the mass concentrations of the alumina nitrate additive (i.e., with increasing values of the target mass concentration of the alumina nanoparticles). The presence of aluminum elements in these ridge-shaped microstructures are confirmed by EDS analysis (see Supplementary Material S7 for the detailed EDS characterization results). Another interesting observation is that the fraction of the total area occupied by these ridge microstructures is significantly greater than the target mass concentration of the nanoparticles. For example, for the nanofluid sample with target mass concentration of nanoparticles of 1.5%, more than 50% of the area in the SEM image is occupied by the ridge-shaped microstructures. This feature indicates that the alumina nanoparticles only contribute to a small fraction of the ridge-shaped structures observed in the SEM images. Instead, the main component of these ridge-shaped structures is a secondary nanostructure formed in between the alumina nanoparticles in the base molten salt.

The morphologies of these secondary nanostructures are better revealed at higher values of magnification (~40,000×). As shown in the figure, these secondary nanostructures appear as a porous foam (percolation network) in the solar salt nanofluid samples. The alumina nanoparticles are not apparent in these SEM images which suggests that the nanoparticles are fully enveloped by the induced secondary nanostructures. The size of the stem (i.e., length) of these nanostructures was estimated to be ~50 nm from the SEM images obtained at higher magnification (see Supplementary Material S8 for the detailed analysis). With increasing values of the target mass concentration of the nanoparticles, the percolation networks in the SEM images—obtained at higher magnification—were observed to be denser (i.e., regions with the amorphous phase intervening the percolation networks was observed to decrease). This is indicative of the merger and aggregation of the secondary nanostructure induced by the nanoparticles. This is probably due to higher nucleation density of the alumina nanoparticles at higher concentration of the aluminum nitrate nonahydrate additive. This could also lead to higher propensity for agglomeration and precipitation of the nanoparticles at higher mass concentrations (i.e., for mass fractions exceeding 1%). Hence, the level of enhancement of the specific heat capacity was reduced for the solar salt nanofluid samples with target mass fractions of nanoparticles exceeding 1%.

## 4. Discussion

### 4.1. Mechanistic Understanding of Specific Heat Capacity Enhancement—The Role of Compressed Layer at Nanoparticle Surface

**Figure 6:**
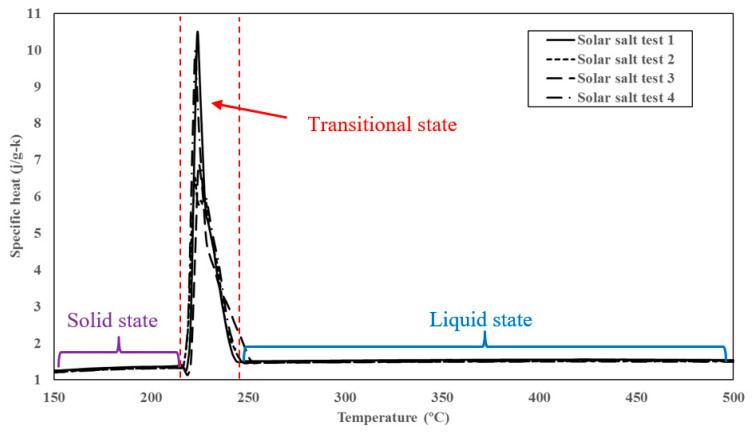
The specific heat capacity curve of solar salt during the phase change process.

In traditional mixing models, it is assumed that the specific heat capacity of a mixture (such as, a nanofluid) is a mass-fraction weighted sum of the specific heat capacity of the individual components (i.e., both the solvent phase and the constituent nanoparticles):

(9) $$C_{total}=\frac{\left[MxC_{n}\right]+\left[\left(M-Mx\right)C_{l}\right]}{M}$$

where, *M* is the total mass of the mixture, *x* is the mass fraction of the nanoparticles, and *C* is the specific heat capacity of the nanoparticle. The subscripts *n* and *l* denote the properties of the bulk liquid phase and nanoparticle, respectively. Typically, solids (and therefore nanoparticles) have significantly lower values of specific heat capacity (less than 1 J/g·K) compared to that of liquids, such as molten salts (e.g., in the range of 1~2 J/g·K). Equation (9), therefore, would predict the degradation in the values of specific heat capacity of solvents when mixed with solids, especially where the solid phase is immiscible or does not form a colloidal suspension. However, experimental results contradict this prediction where specific heat capacity values are enhanced on mixing with solid nanoparticles (e.g., in the form of stable colloidal suspension). Modified models were, therefore, developed to mitigate these discrepancies by accounting for the high surface energy of nanoparticles compared to the bulk property values of the solid phase [48,49] and recognizing the existence of a “compressed phase” or “compressed layer” that is induced on the surface of the nanoparticle by molecules of the solvent phase that are adsorbed on the surface [50,51]. In the compressed layer, the dominance of the adhesive forces over the cohesive forces causes the molecules of the solvent phase in the vicinity of the nanoparticle surface to be packed into smaller volumes which results in a higher mass density and a greater total potential energy. If we segment the total internal energy into the kinematic energy (“KE”, resulting from translational motion of the constituent molecules) and the potential energy (“PE”, resulting from the relative position of the constituent molecules), then the ratio between PE and KE for molecules confined in the compressed layer can be increased by up to 10 times that of the molecules in the bulk phase of the solvent (see Supplementary Material S9 for detailed quantitative analysis). Thus, a larger amount of total energy (KE + PE) input is required for increasing the KE of the molecules in the compressed phase (i.e., to the same level as the KE of the molecules in the bulk phase of the solvent). Since temperature is a direct indication of KE, this analysis essentially implies that the molecules in the compressed layer have higher values of specific heat capacity compared to that of the bulk phase of the solvent. In other words, it becomes more difficult for the molecules in the compressed layer to gain momentum, unless the energy input is large enough to overcome the potential energy barrier of the compressed phase. This scenario closely resembles the melting process, in which a significant fraction of the energy input is expended in overcoming the intermolecular bonds in the solid phase (while a very small fraction of the total energy is available for increasing the kinematic energy of each individual molecule in the solid phase as it transitions to the liquid phase). Hence, if we assume that the inter-molecular structure of the compressed phase is similar to the solid phase of the pure solvent (i.e., the molten salt or pure solar salt) at the phase transition point from solid to liquid, then the specific heat capacity of the compressed phase can be estimated to be similar to that of the solid phase near the melting point. Figure 6 shows that the specific heat capacity of pure molten salt measured by modulated differential scanning calorimetry (MDSC) over four continuous cycles. According to the results shown in the figure, the specific heat capacity of the compressed phase (as shown by the peak value of the specific heat capacity curve at the melting point) is approximately 10 times that of the liquid phase.

If the specific heat capacity of the compressed layer adsorbed on nanoparticle surface is to be included as a distinctly separate component for predicting the specific heat capacity of molten salt nanofluids, then the net value of the specific heat capacity of molten salt nanofluid is calculated by adding the mass fraction weighted values of the specific heat capacity of the three constituents—the nanoparticle, the compressed phase and the bulk liquid phase (solvent)—as shown below:

(10) $$C_{total}=\frac{\left[MxC_{n}\right]+\left[\frac{m_{s}}{m_{n}}MxC_{s}\right]+\left[\left(M-Mx-\frac{m_{s}}{m_{n}}Mx\right)C_{l}\right]}{M}$$

where, (*m_s_/m_n_*) is the ratio between the mass fraction of the compressed layer and the nanoparticle and $C_{s}$ is the specific heat capacity of the compressed layer. The calculation of this ratio can be performed using the shape and size (i.e., morphology) of the nanoparticle. As an example, assume that the compressed layer forms a concentric envelop with a thickness of *δ* around a spherical nanoparticle with a diameter of *D_np_*, then Equation (10) can be re-written as:

(11) $$C_{total}=\left[x\right]C_{n}+\left\{\left(x\frac{\rho_{s}}{\rho_{n}}\right)\left[{\left(1+\frac{2\delta }{D_{np}}\right)}^{3}-1\right]\right\}C_{s}+\left\{1-x-\left(x\frac{\rho_{s}}{\rho_{n}}\right)\left[{\left(1+\frac{2\delta }{D_{np}}\right)}^{3}-1\right]\right\}C_{l}$$

**Figure 7:**
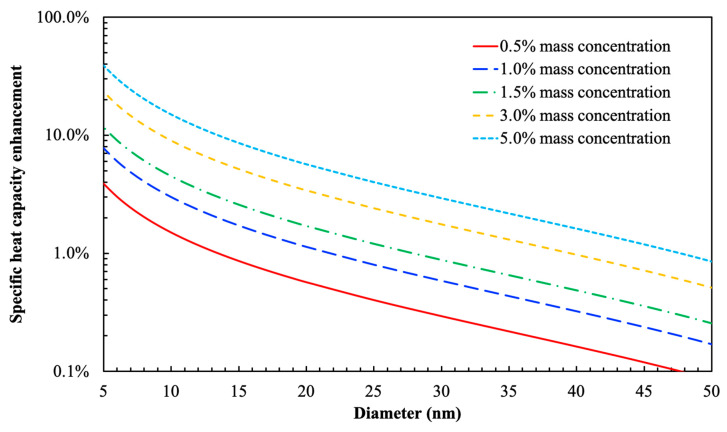
Numerical prediction for the percentage of specific heat capacity enhancement of solar salt nanofluid (containing Al_2_O_3_ nanoparticles) as a function of nanoparticle size at five different mass concentrations.

As predicted by Equation (11), when diameter of the nanoparticle is much greater than the thickness of the compressed layer, the volume fraction of the compressed layer on nanoparticle surface will become sufficiently small such that the proportional contribution to the net value of specific heat capacity will be negligible or insignificant. According to the past literature study, the thickness of the compressed layer absorbed on nanoparticle surface is around 1 nm [52]. The specific heat capacity and density of pure solar salt (i.e., NaNO_3_-KNO_3_ eutectic) were measured to be 1500 J/kg·K and 2000 kg/m^3^, respectively [53]. The specific heat capacity and density of the Al_2_O_3_ nanoparticles were estimated to be 880 J/kg·K and 3890 kg/m^3^, respectively, according to US Research Nanomaterials, Inc. Substituting these parameters in Equation (11), we can predict the specific heat capacity of molten salt nanofluid containing different mass concentrations of nanoparticles, as shown in Figure 7.

As shown by the results in Figure 7, the level of enhancement is highly sensitive to the mass concentration and the diameter of the nanoparticles. In particular, the specific heat capacity is only enhanced by a significant margin when the mass concentration of the nanoparticle is sufficiently large and the size of the nanoparticle is sufficiently small. However, for the three mass concentrations explored in this study, the predictions in Figure 7 only shows marginal change (<1%) in the value of the specific heat capacity and the effect of the compressed phase (i.e., the contribution of the compressed phase to the net specific heat capacity) is almost negligible when the size of the nanoparticle is greater than 25 nm. This prediction is, therefore, inconsistent with the experimental results obtained in this study (as well as prior reports in the literature)—since significant enhancement in specific heat capacity values were observed for solar salt nanofluids with alumina nanoparticles at mass concentration of 1% and for the diameter of the nanoparticles ranging from 10~50 nm. In other words, the contribution from the compressed layer itself is insufficient for explaining the substantial enhancement in the specific heat capacity.

### 4.2. Mechanistic Understanding of Specific Heat Capacity Enhancement—The Role of Secondary Nanostructures

**Figure 8:**
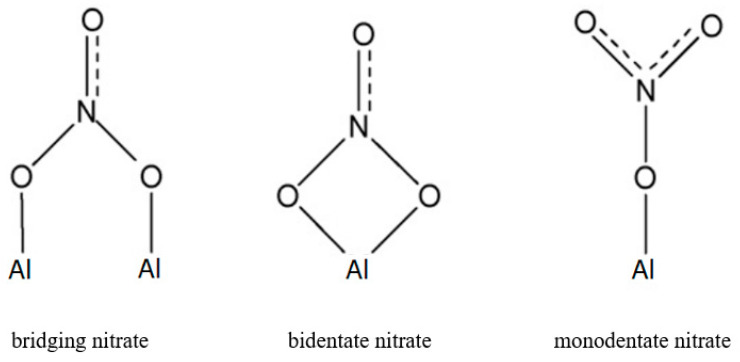
Possible chemisorption structure of nitrate ion on Al_2_O_3_ surface.

As stated earlier, an important feature observed from the SEM images in Figure 5 is that the fraction of area occupied by the ridge-shaped microstructures is far greater than the mass concentration of the nanoparticle in the molten salt. This characteristic essentially suggests that the presence of the nanoparticle may induce a long-range effect in the molten salt which facilitates the formation of secondary micro/nano structures. Molten salts are ionic liquids which typically dissociate into positive and negative ions. In the liquid state, the solar salt is composed of free Na^+^, K^+^ and ${\mathrm{NO}}_{3}^{-}$ ions. Due to the exposed crystal facets and surface defects on a solid surface (such as a nanoparticle surface), these ions can be adsorbed preferentially depending on the intermolecular affinity for each ion for a given surface. The preferential adsorption of ions on a solid surface significantly alters its apparent surface charge distribution resulting in accumulation of surface charges. Considering Al_2_O_3_ nanoparticle as an example, the ${\mathrm{NO}}_{3}^{-}$ anion could be chemisorbed to the metal cation in the particle surface with three different bonding structures [54], as shown in Figure 8. In recent years, the importance of ionic interaction on the specific heat capacity of molten salt nanofluid has been acknowledged by a number of studies. For example, Mondragón et al. [55] performed Fourier transform infrared spectroscopy tests to confirm the chemisorption of nitrate ions on silica and alumina nanoparticles in molten salt nanofluids. They further measured the ionic exchange capacity (IEC) of the nanoparticles and observed a positive relationship between the specific heat capacity enhancement of the nanofluid and the IEC of the nanoparticle. Such a finding confirms the contribution of absorbed ions on the specific heat capacity of molten salt nanofluids.

**Figure 9:**
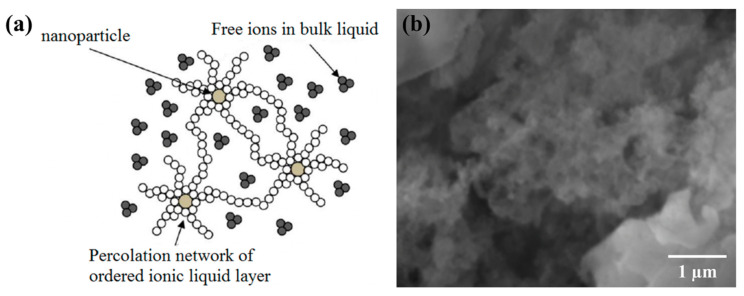
Formation of secondary nanostructure (percolation network) by extended ordering of molecules from the chemisorbed anions on the surface of nanoparticles. (**a**) Schematic showing the secondary nanostructures forming a percolation network between nanoparticles from free ions (or molecules) in the bulk phase of the solvent. (**b**) SEM image showing percolation network formed by secondary nanostructures in solar slat nanofluids with alumna nanoparticles.

The preferential adsorption of ${\mathrm{NO}}_{3}^{-}$ on the surface of the alumina nanoparticle causes progressive build up of a net negatively charged surface potential (i.e., the nanoparticle develops a negative charge with reference to the bulk solvent phase). This in turn, induces substantial electrostatic attractive/repulsive forces near the particle surface as well as between nanoparticles in the vicinity of each surface. Various reports in the literature have demonstrated that under the influence of a strong electrostatic driving force, the ordered ionic liquid layer could extend to more than 10 nm in thickness [56,57,58]. In certain cases, the ordered layer extends 1000 nm, in which strong preferential alignments of molecules were revealed in the compressed layer where the adsorbed molecules can align mutually to start mimicking the underlying crystal structure of the solid surface (i.e., an epitaxial structure can form) [59]. Therefore, it is inferred that the mutual interactions in multi-body and multi-component systems can cause these ordered layers to extend from the surface of each nanoparticle and form “bridges” to other nanoparticles in the vicinity. These bridges (i.e., the secondary nanostructures) will then cause the nanoparticles to become inter-connected and to form the foam-like percolation networks, as shown in Figure 9. Similar types of feature have also been observed and confirmed in our previous studies [16,60].

Considering that long-range secondary structures are observed in the solar salt nanofluid samples (as shown in Figure 9), Equation (10) could be modified to predict the net heat capacity of molten salt nanofluids by incorporating the additional effect arising from the long-range nanostructures induced in the molten salt, as follows:

(12) $$C_{total}=\frac{\left[MxC_{n}\right]+\left[\frac{m_{s}}{m_{n}}MxC_{s}\right]+\left[\frac{m_{f}}{m_{n}}MxC_{f}\right]+\left[\left(M-Mx-\frac{m_{s}}{m_{n}}Mx-\frac{m_{f}}{m_{n}}Mx\right)C_{l}\right]}{M}$$

where, *m_f_*/*m_n_* is the ratio of mass fraction between induced long-range nanostructures and nanoparticle, while *C_f_* is the heat capacity of the long-range nanostructure. The determination of *m_f_*/*m_n_* and *C_f_* would require some additional technique and exploration in the future. In particular, if the secondary nanostructures are indeed composed of ordered ionic layers formed under the electrostatic driving force, then these nanostructures can have different elemental composition and structure from the bulk amorphous phase which make it more difficult to estimate their thermophysical properties. Nevertheless, since the mass fraction of the secondary nanostructures can be quite significant, Equation (12) provides a potentially reasonable explanation to rationalize the significant specific heat enhancement in molten salt nanofluid that is achieved at only a minute concentration of nanoparticles. Similar type of model has also been proposed in the study by Reinhard Hentschke who introduced the “interacting mesolayer” concept to account for the contribution from the long-range “solid-like” layer structure formed in the molten salt nanofluid. While his model can also yield quite consistent specific heat capacity enhancement with some experimental studies when using properly selected parameters, the specific heat capacity of the long-range mesolayer structure remains unclear which restricts the application of the model. Therefore, the endeavor to estimate the specific heat capacity of nanofluids, particularly for molten salt nanofluids, needs to account for the material property values as well as the molecular structure of the solvent molecules in both the compressed phase and the secondary nanostructures that can be induced by the presence of the nanoparticles in the solvent medium. It is possible that the chemical composition and molecular level ordering of the compressed phase and secondary nanostructures can be very different from each other as well as from the bulk phase of the solvent.

### 4.3. Mechanistic Understanding of the Effect of Nanoparticle Concentration on Specific Heat Capacity Enhancement

**Figure 10:**
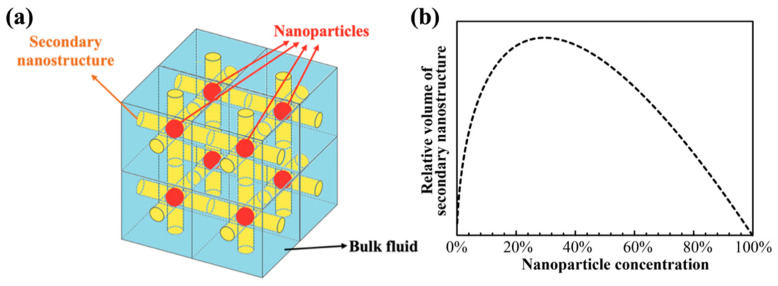
(**a**) Conceptual model of percolation network formed by the secondary nanostructures (in yellow) between adjacent nanoparticles (in red) in a nanofluid sample (the bulk of the solvent phase is in blue color). The diameter of the nanoparticles and the percolation network are shown to be of the same fixed size. (**b**) Volume fraction of secondary nanostructures as a function of the mass fraction of the nanoparticles (of a fixed size and distributed uniformly in the volume of the nanofluid sample).

In a nanofluid sample (i.e., a stable nanoparticle colloidal suspension), the average inter-particle distance shrinks with increasing mass fraction of the nanoparticles (for a fixed size of the nanoparticles). If the secondary micro/nano structures are confined to the space between the nanoparticles, then the mass fraction of the secondary nanostructures should be reduced with decreasing distance between the nanoparticles. Considering an ideal scenario in which all nanoparticles of the same size are distributed uniformly in a molten salt nanofluid sample and all the percolation network formed by the secondary structures of a fixed diameter (say, in the form of cylindrical shape of a fixed diameter) are confined to the shortest distance between any two adjacent nanoparticles (as shown in Figure 10a), then the volume of the secondary nanostructures ($V_{secondary}$) is proportional to the inter-particle distance ($A_{inter-particle}$) and mass fraction of the nanoparticles ($\varphi_{np}$), as shown in the following equation:

(13) $$V_{secondary}\propto A_{inter-particle}\cdot \varphi_{np}$$

Assuming homogeneous distribution of the nanoparticles of a fixed size, the inter-particle distance is negatively correlated to nanoparticle concentration as:

(14) $$A_{inter-particle}\propto \frac{1-\varphi_{np}^{1/3}}{\varphi_{np}^{1/3}}$$

Substituting Equation (14) in Equation (13) yields:

(15) $$V_{secondary}\propto \varphi_{np}{}^{2/3}\left(1-\varphi_{np}{}^{1/3}\right)$$

Based on Equation (15), the variation of the volume of the secondary nanostructure with nanoparticle concentration (mass fraction) is plotted in Figure 10b. The plot shows that the volume fraction of secondary nanostructure starts to decline when nanoparticle concentration exceeds 30%.

**Figure 11:**
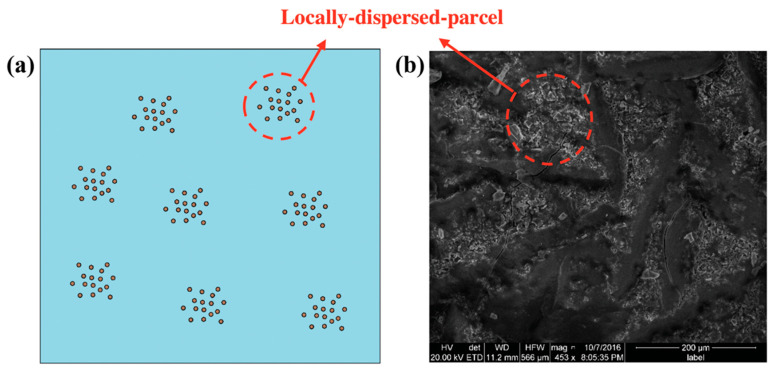
Heterogeneous distribution of nanoparticles in a molten salt nanofluid sample showing locally-dispersed nanoparticles in each parcel and the parcels are dispersed throughout the sample in a heterogeneous configuration. (**a**) Schematic diagram. (**b**) SEM image of solar salt nanofluid sample obtained by one-step synthesis protocol from aluminum nitrate nonahydrate additive (with a target mass fraction of 1% for alumina nanoparticles).

In reality, the nanoparticles are not distributed uniformly in the molten salt nanofluid samples. As shown by the SEM images in Figure 5, the nanoparticles tend to cluster into closely packed ensembles (or parcels) and each of these parcels are dispersed throughout the volume of the nanofluid samples in discrete groups. Secondary nanostructures are observed to form between the nanoparticles in each parcel. However, secondary nanostructures are not generally apparent between different parcels. Therefore, when the nanoparticles are inter-connected by these secondary nanostructures in a parcel, they tend to be isolated from another parcel due to the steric effect. Such an effect prevents the nanoparticle from being uniformly dispersed in the molten salt solvent at liquid state. This locally dispersed parcel configuration, as shown in Figure 11, results in a higher value of effective nanoparticle concentration in each discrete parcel (i.e., the local values of mass fraction in each of these parcels are significantly higher than that of the global average value). Consequently, the optimum value (i.e., the global average value) of the mass concentration of the nanoparticles is achieved at ~1% while the local value of mass fraction for the nanoparticles (in a parcel) is probably in the vicinity of ~30%, as predicted in Figure 10b. In addition, it can be observed in Figure 5 that as the global average value of the mass fraction of the nanoparticles is increased from 0.5% to 1.5%, the void space between the nanoparticles in a parcel is reduced significantly. Hence, from a purely geometric consideration it is suggested that the optimal value of the mass fraction of the nanoparticles for maximizing the volume (or mass fraction) of the secondary nanostructures is in the range of 0.5% to 1%.

## 5. Conclusions

In this study, solar salt-Al_2_O_3_ nanofluids at three different concentrations were prepared by a one-step method in which the oxide nanoparticles were generated in the salt melt directly from precursors. The morphological structures of the obtained nanomaterials were examined under scanning electron microscopy and the specific heat capacities were measured using the temperature history (T-history) method. Electron microscopy revealed clustering of Al_2_O_3_ nanostructures in closely packed ensembles which were dispersed throughout the volume of the nanofluid samples in discrete groups. A non-linear enhancement in the specific heat capacity of molten salt nanofluid was observed from the thermal characterization at a nanoparticle mass concentration of 0.5%, 1.0%, and 1.5%. In particular, a maximum enhancement of 38.7% in specific heat was found for the nanofluid sample prepared with a target nanoparticle mass fraction of 1.0%. According to the morphological characteristic of the molten salt nanofluid sample observed in SEM images, it is hypothesized that the chemisorption of free ions on the nanoparticle surface and the associated long-range ionic effect could induce the formation of secondary nanostructures in the solvent phase of molten salt nanofluid. These secondary nanostructures are believed to play a critical role in controlling the enhancement in the specific heat capacity of the molten salt nanofluids. These secondary network structures are inherently an extension of the compressed layer formed in the vicinity of the nanoparticle surface and can occupy a significant fraction of the total volume in the molten salt nanofluid sample even at a very low nanoparticle concentration. Hence, the contribution from these secondary nanostructures, which acts virtually as the fourth phase in the molten salt nanofluid system (i.e., in addition to the bulk solvent, the nanoparticle, and the compressed layer on the nanoparticle surface), should be taken into consideration when analyzing the specific heat capacity enhancement in the molten salt nanofluid system. Finally, using a simple mathematical model, it was shown that for a constant value and uniform size of the nanoparticles the volume fraction of the third phase (i.e., these networks of secondary nanostructures forming the percolation network between adjacent nanoparticles) can first increase and then decline with increasing nanoparticle concentration. Such physical behavior implies that an optimal value exists for nanoparticle concentration for maximizing the enhancement of the specific heat capacity of molten salt nanofluid samples.

Fundamentally, there are still many unknowns that need to be resolved in order to elucidate the transport mechanisms that are responsible for the anomalous enhancement of specific heat capacity and thermal conductivity of molten salt nanofluid samples. One critical issue is the knowledge of how the ions are arranged/packed in the secondary nanostructures (compressed phase as well as the percolation network), their nucleation, growth and assembly. The structure-property relationships for these secondary nanostructures need to be modeled and validated experimentally. Studies in the literature have alluded to the “ordering behavior” of room temperature ionic liquids in the vicinity of a charged surface. However, similar studies for high temperature molten salt (>500 °C), involving both experimental and numerical approaches, are still unavailable and are desired in future. Sophisticated instrumentation that operates at these high temperatures needs to be developed in order to develop enhanced cognition of the transport processes involved in the molten salt nanofluid samples. From the practical perspective, ensuring a good stability of the nanofluid is also an important issue since the properties of nanofluids could be drastically affected by the clustering and aggregation of nanoparticles. However, keeping nanoparticles suspended uniformly in the base fluid for long enough is still a challenging task, since clustering of nanoparticles in fluid is a natural and spontaneous process due to the strong Brownian motion of liquid molecules which promote collision between nanoparticles, while the high surface energy of nanoparticles promotes adhesion after collisions. For conventional nanofluids based on water or organic solvents, some of the typical strategies utilized for ensuring long-term stability include mechanical mixing (e.g., ultrasonication, high-pressure homogenizer), adding dispersing agents, controlling the pH value of the solvent, and grafting functional groups on the surface of nanoparticles [43]. For a molten salt system, however, the adsorption of free nitrate ions on the nanoparticle surface naturally provides a strong electrostatic repulsive force between different nanoparticles which reduces the possibility of agglomeration. Developing a quantitative relationship between the ion adsorption to the surface and the stability of molten salt nanofluid will also be an important topic to explore in the future.

## Figures and Tables

**Table 1:** Mass of raw materials used in the synthesis.

	Raw Material Mass (g) for Synthesis			Final Product Mass (g)		
Target Nanoparticle Concentration	NaNO_3_	KNO_3_	Al(NO_3_)_3_·9H_2_O	Solar Salt	Al_2_O_3_	Total
0.5%	20.895	13.93	1.287	34.825	0.175	35
1.0%	20.790	13.86	2.575	34.650	0.350	35
1.5%	20.685	13.79	3.862	34.475	0.525	35

**Table 2:** The mass fraction of precursor used and the target mass fraction of alumina nanoparticles, are: (top table) 3.5% and 0.5%; (middle table) 6.9% and 1%; and (bottom table) 10.1% and 1.5%, respectively.

**Specific heat enhancement for solar salt nanofluid containing 0.5% alumina nanoparticles**					
Test number	300 °C	400 °C	500 °C	Average	STD
1	37.9%	17.6%	11.4%	20.8%	7.0%
2	28.2%	13.2%	9.7%	15.9%	6.0%
3	42.5%	18.1%	11.1%	20.8%	8.0%
4	26.3%	11.3%	6.6%	13.6%	5.1%
5	39.7%	19.5%	13.1%	22.1%	7.2%
6	39.3%	15.8%	8.5%	18.9%	7.9%
7	30.6%	16.6%	10.1%	17.7%	6.2%
8	28.1%	11.3%	6.0%	13.8%	6.3%
9	32.0%	12.5%	7.6%	15.3%	7.2%
10	36.0%	13.9%	7.0%	15.7%	7.8%
Average	34.1%	15.0%	9.1%		
		**Grand Avg**	17.4%	**Grand STD**	7.5%
**Specific heat enhancement for solar salt nanofluid containing 1.0% alumina nanoparticles**					
Test number	300 °C	400 °C	500 °C	Average	STD
1	44.5%	36.0%	28.1%	35.1%	5.1%
2	60.3%	34.7%	30.3%	37.6%	7.7%
3	53.6%	37.0%	31.2%	38.7%	6.7%
4	61.3%	36.9%	32.8%	41.6%	8.6%
5	54.3%	38.1%	34.1%	40.3%	6.0%
6	53.8%	35.5%	30.4%	38.5%	6.9%
7	57.4%	32.6%	28.7%	36.0%	7.5%
8	59.4%	34.7%	32.0%	38.7%	7.5%
9	52.6%	37.3%	31.7%	39.1%	6.4%
10	60.7%	36.2%	32.1%	41.8%	9.2%
Avg	55.8%	35.9%	31.2%		
		**Grand Avg**	38.8%	**Grand STD**	7.5%
**Specific heat enhancement for solar salt nanofluid containing 1.5% alumina nanoparticles**					
Test number	300 °C	400 °C	500 °C	Average	STD
1	46.5%	27.8%	20.2%	29.4%	7.0%
2	67.6%	29.6%	20.5%	32.8%	10.8%
3	53.1%	27.9%	20.3%	30.6%	8.3%
4	58.0%	28.7%	20.6%	31.2%	9.3%
5	52.1%	27.6%	21.6%	30.6%	7.9%
6	73.2%	30.9%	22.4%	35.6%	12.0%
7	58.6%	28.6%	22.3%	32.4%	9.7%
8	64.2%	27.7%	20.1%	32.3%	10.8%
9	56.1%	26.3%	19.6%	30.3%	9.4%
10	57.2%	28.8%	22.2%	32.2%	9.1%
Avg	58.7%	28.4%	21.0%		
		**Grand Avg**	31.8%	**Grand STD**	9.7%

* The temperature measurement is subjected to an uncertainty of ±0.75%.

## Supplementary Materials

The following are available online at https://www.mdpi.com/2079-4991/10/11/2266/s1.
