# Supplementary material for: Synthesis and Characterization of Molten Salt Nanofluids for Thermal Energy Storage Application in Concentrated Solar Power Plants—Mechanistic Understanding of Specific Heat Capacity Enhancement
Source: Nanomaterials (Basel). 2020 Nov 16;10(11):2266. doi: 10.3390/nano10112266 (PMC7697307; doi:10.3390/nano10112266)
Supplement: Supplementary file 1 [file nanomaterials-10-02266-s001.pdf]

Supplementary Materials

Synthesis and Characterization of Molten Salt Nanofluids for Thermal Energy Storage Application in Concentrated Solar Power Plants—Mechanistic Understanding of Specific Heat Capacity Enhancement

Binjian Ma [1,*], Donghyun Shin [2] and Debjyoti Banerjee [3]

1 School of Mechanical Engineering and Automation, Harbin Institute of Technology (Shenzhen), Shenzhen 518055, China
2 School of Engineering & Technology, Central Michigan University, Mount Pleasant, MI 48859, USA; shin1d@cmich.edu
3 Department of Mechanical Engineering, Department of Petroleum Engineering, Mary Kay O'Connor Process Safety Center, Texas A&M University, College Station, TX 77843, USA; dbanerjee@tamu.edu
* Correspondence: mabinjian@hit.edu.cn

S1. The Thermogravimetric Analysis (TGA) of Different Candidate Precursors for the Generation of Nanoparticles In Situ in the Molten Salt Environment

A total of 18 different salt hydrate and metal compounds were tested by the thermogravimetric analysis (TGA) in a flux of nitrogen at 100 mL/min with a ramping rate of 5 °C/min (using SDT Q600 TA Instrument). The corresponding decomposition curves of each chemical are summarized in Figure S1. Among all chemicals, the decomposition of aluminum nitrate nonahydrate was found to progress most rapidly with increasing temperature during the heating cycle. In other words, the decomposition reaction of aluminum nitrate nonahydrate is highly active even in the low temperature range (e.g., <250 °C). The low decomposition temperature of aluminum nitrate nonahydrate makes it the most favorable as a candidate precursor for generating monodispersed nanoparticles, since the crystal growth and coagulation processes are less drastic at low temperature conditions. Furthermore, aluminum nitrate nonahydrate shares the same anion with the molten salt solvent which implies that no exterior contamination (e.g., chloride ions) is explicitly introduced into the molten nitrate salt system. Therefore, aluminum nitrate nonahydrate $Al(NO_3)_3{\cdot}9H_2O$ is finally selected as the target precursor in this study for the synthesis of molten salt nanofluid.

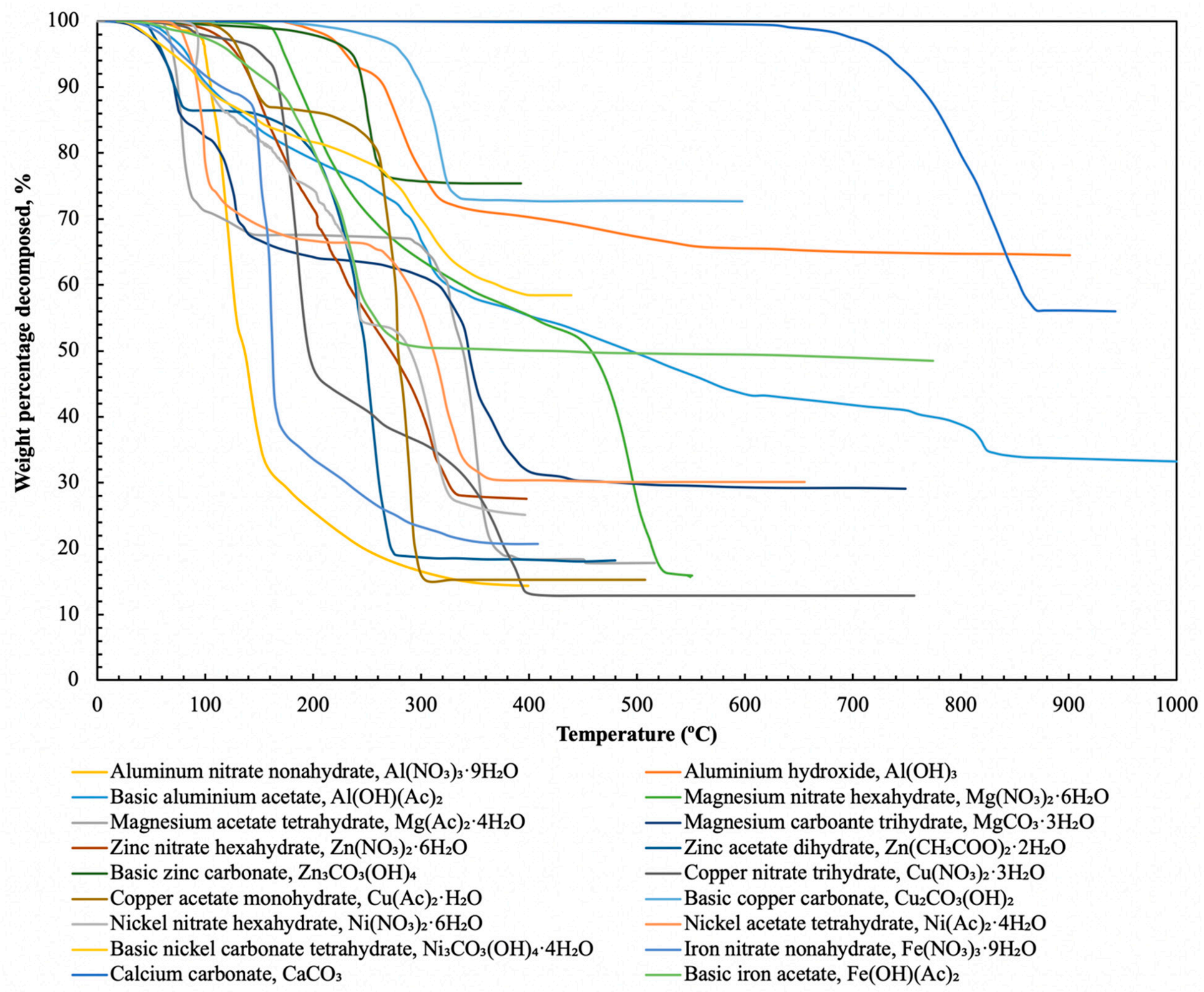

Figure S1. The thermal decomposition curve of different chemicals obtained from the thermogravimetric analysis (TGA).

S2. Fin Efficiency of the Glass Disk Beneath the Molten Salt Test Vials

During the T-history test, the test tubes are supported by the glass disk. Therefore, the heat will be transferred to the test tube via two paths as shown in Figure S2. ***Path 1*** only involves the convective heat transfer between air and test tube. The conduction and convection across the circular disk in ***Path 2*** can be treated as the annual fin problem as shown in Figure S3 [1]. In our study, we have r_1 = 1.77 cm, r_2 = 5.00 cm, t = 0.15 cm, and L = 3.31 cm. The glass disk has a thermal conductivity of k = 0.8 W/(m·K). With these parameters and a natural convection heat transfer coefficient of 5 W/(m^2·K), we have $L_c^{3/2}(h/kA_p)^{1/2}$ = 2.13 and r_{2c}/r_1 = 2.87. From Figure S3, we can find that the fin efficiency of the circular disk is around η_f = 25%.

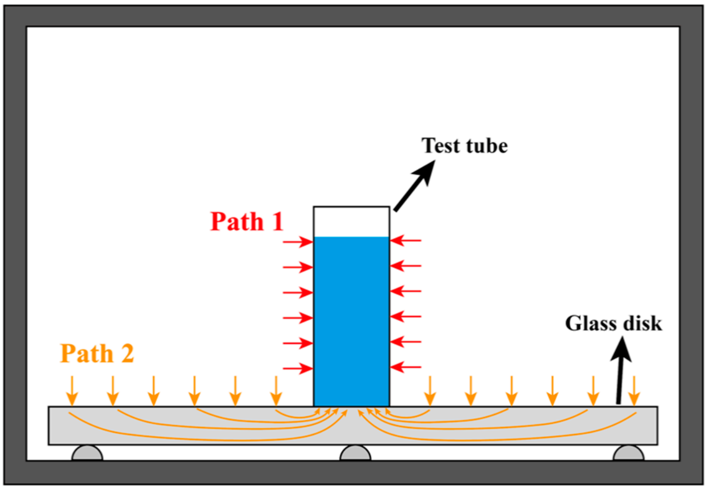

Figure S2. The two different paths of heat transfer from the air to the test tube.

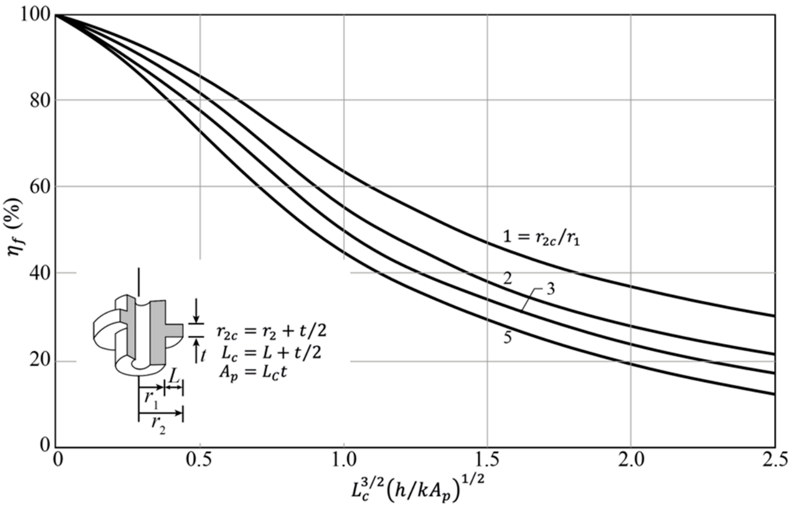

Figure S3. Efficiency of annular fins of rectangular profile [1].

S3. High-Temperature Evaporative Mass Loss Test of Molten Salt and Nanofluid Samples

The temperature histories of reference material (water) and testing liquids (propylene glycol and isopropyl alcohol) in repeated heating and cooling cycles are shown in Figure S4. It was noticed that the change of ambient temperature comprises two separate stages with distinct slope change. This is corresponding to the point where the thermostat of the environment chamber reaches a setpoint value of 60 °C. The heating/cooling fan stopped working afterwards and the temperature change inside the chamber was primarily driven by thermal diffusion. Nevertheless, it was shown in all testing cycles that propylene glycol had the fastest thermal response to the ambient environment temperature change comparing to water and isopropyl alcohol. This indicates that propylene glycol has the smallest thermal inertial among all three samples. Considering that these liquids have similar density, the specific heat capacity of the three samples follows the sequence of $c_{p,\mathrm{PG}} < c_{p,\mathrm{IPA}} < c_{p,\mathrm{water}}$.

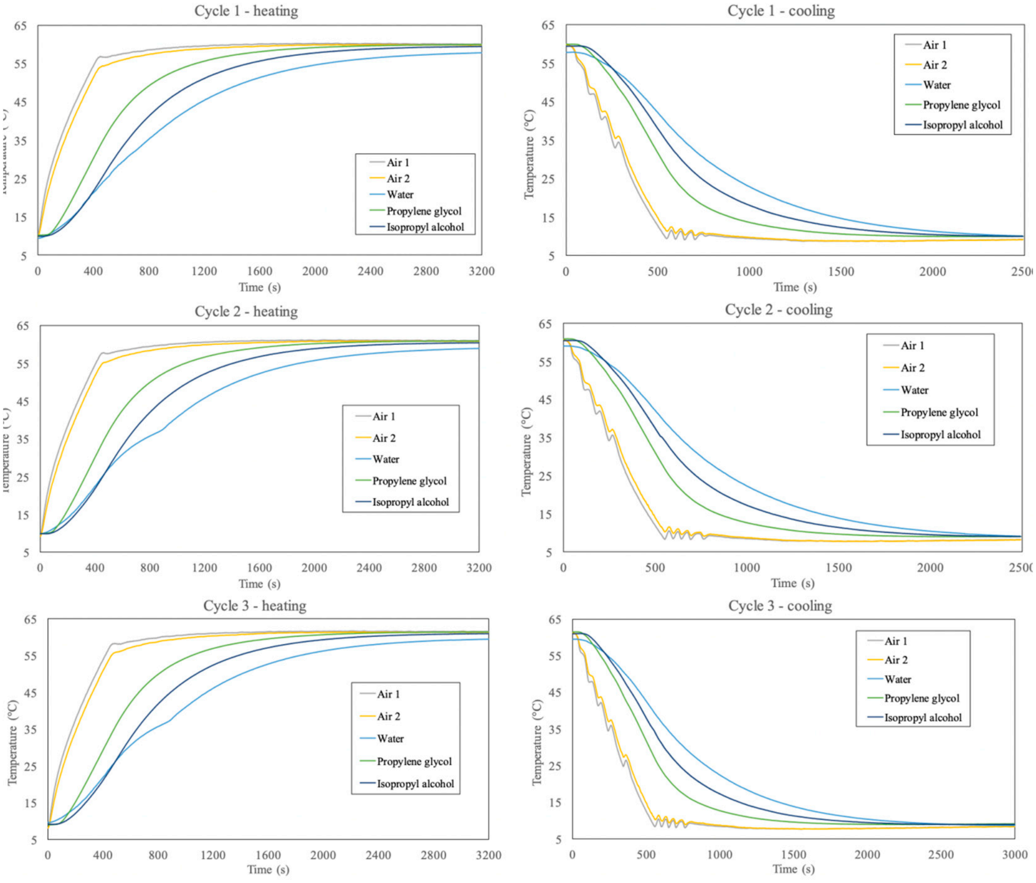

Figure S4. Temperature responses of different testing liquids in repeated T-history experiments at room temperature.

Figure S5 and Table S1 show the ratio of specific heat capacities between the two testing liquids and water obtained from the T-history tests. The result was calculated from in the temperature range from 20 °C to 50 °C. It was found that the measurements from heating cycles generally yield higher specific heat capacity values compared to that from cooling cycles. Nevertheless, the results showed that the average specific heat capacity of propylene glycol and isopropyl alcohol are 53% and 67% of that of water, which corresponds to 2.24 J/(g·K) and 2.80 J/(g·K), respectively. Such values closely match the literature measurements [2,3], which suggests that the transient T-history method is applicable for measuring specific heat capacity of normal liquid with high accuracy.

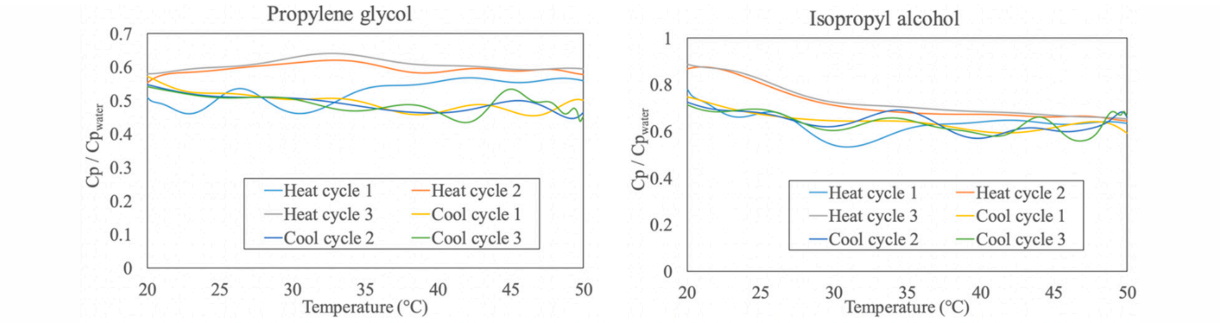

Figure S5. Specific heat capacity ratio as a function of temperature (obtained from T-history experiments) between testing liquids and reference sample (water).

Table S1. Comparison between T-history measurements of specific heat capacity of propylene glycol and isopropyl alcohol and literature values.

	Specific heat capacity ratio over water (evaluated from 20 °C to 50 °C)		Absolute specific heat capacity value [J/(g·K)]	Comparison with literature measurements	
	**Average value**	**Error (percentage)**		**Literature values [J/(g·K)]**	**Error (percentage)**
Propylene glycol	53.5%	10.3%	2.24 (± 0.23)	2.33 [2]	3.8%
Isopropyl alcohol	66.7%	10.0%	2.80 (± 0.28)	2.71 [3]	3.3%

S4. High-Temperature Evaporative Mass Loss Test of Molten Salt and Nanofluid Samples

To determine the evaporative mass loss of the molten salt and nanofluid samples, an array of solar salt samples (with and without nanoparticles) with a mass close to 30 g were held at 550 °C for 202.5 h inside a furnace, as shown in Figure S6. The total mass of the samples (including container) were recorded every 67.5 h. By calculating the mass change of each sample before and after the heating cycle, we then determined the evaporative mass loss rate of the samples per hour. Table S2 shows the experimental test results. As shown in the table, the average evaporative mass loss of pure molten salt and molten salt nanofluid sample is less than 1% per hour. In our study, the duration of the T-history test was less than 1250 s (<0.34 h) which suggests that the percentage mass loss of the sample would be less than 0.3%. In addition, the temperature of the test samples was increased gradually from 275 °C to 550 °C instead of being kept at 550 °C during the entire test. Therefore, the evaporative mass loss of the sample would be even smaller. Considering these factors, we concluded that the evaporative mass loss of the molten salt and nanofluid samples during the T-history is negligible and does not have a significant effect on the specific heat capacity measurement results.

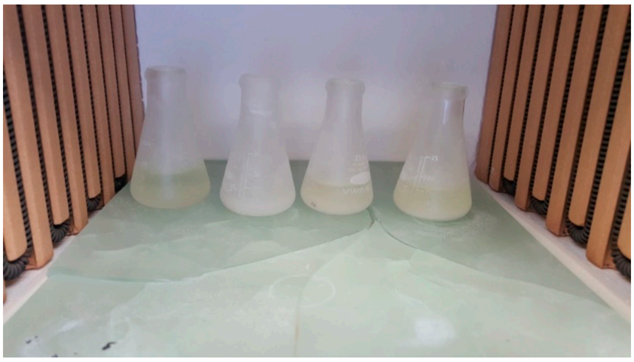

Figure S6. The experimental setup of high-temperature evaporative mass loss test.

Table S2. The evaporative mass loss data of pure solar salt and solar salt nanofluid sample over 202.5 h at a temperature of 550 °C.

**Mass change of test samples during the evaporative mass loss test (g)**				
	t = 0 h	t = 67.5 h	t = 135 h	t = 202.5 h
Pure solar salt	28.636	20.417	9.358	0.119
Solar salt nanofluid	30.434	19.990	3.578	1.398
**Percentage mass loss per hour**				
	1st heating period (0~67.5 h)	2nd heating period (67.5~135 h)	3rd heating period (135~202.5 h)	Average
Pure solar salt	0.43%	0.80%	1.46%	0.90%
Solar salt nanofluid	0.51%	1.22%	0.90%	0.88%

S5. The repeated T-history tests of solar salt nanofluid samples obtained by adding $Al(NO_3)_3{\cdot}9H_2O$ precursor for yielding Al_2O_3 nanoparticles

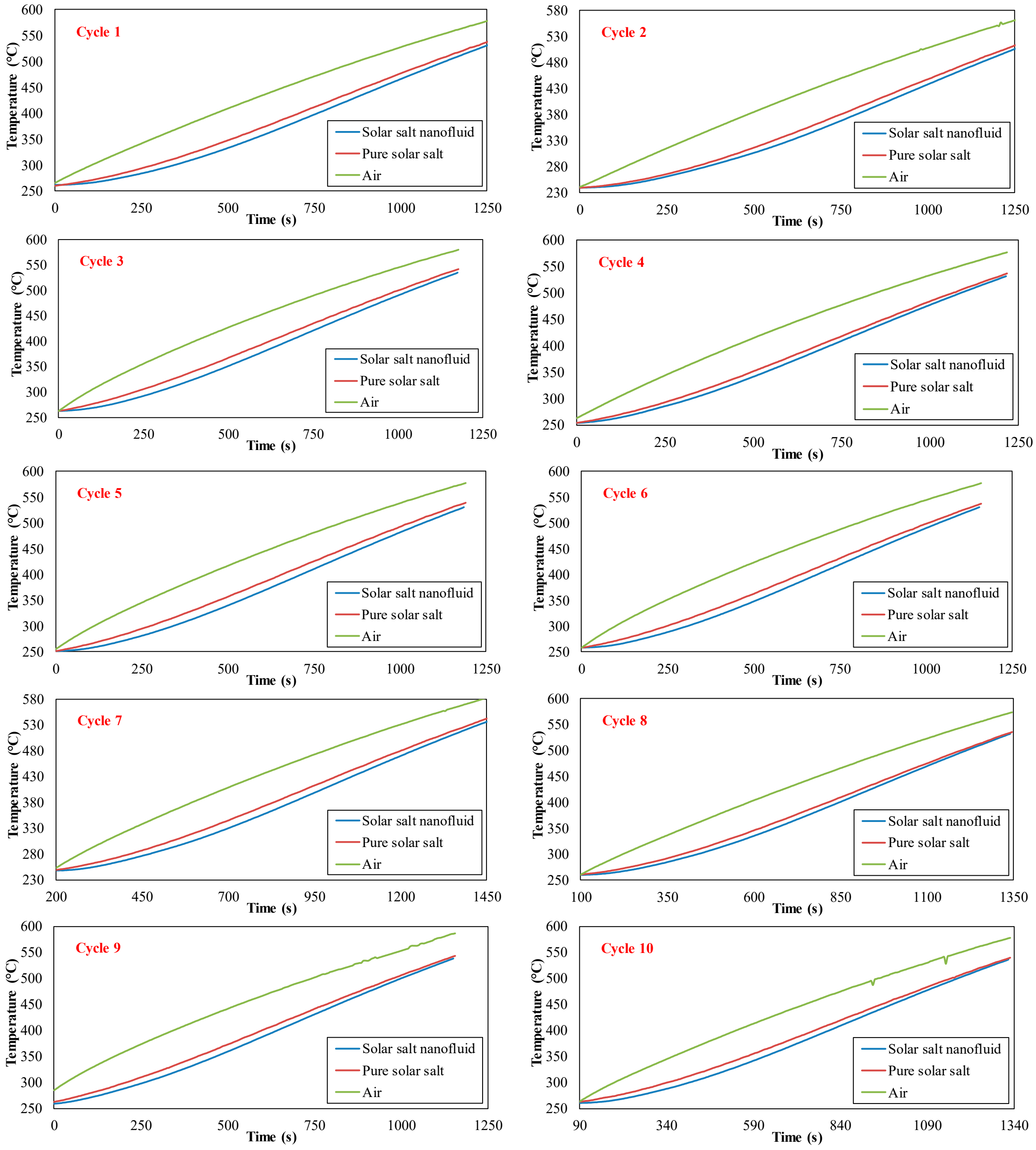

Figure S7. Plot of temperature response obtained from T-history experiments for thermocouples places in air (in the furnace), pure solar salt samples and solar salt nanofluid samples (prepared using one-step synthesis protocol). The molten salt nanofluid samples were obtained by adding $Al(NO_3)_3{\cdot}9H_2O$ at 3.5% mass fraction (as nanoparticle precursors for yielding nanofluids) to pure solar salt for a target mass fraction of 0.5% for the resulting Al_2O_3 nanoparticles obtained by thermal decomposition (one-step synthesis protocol).

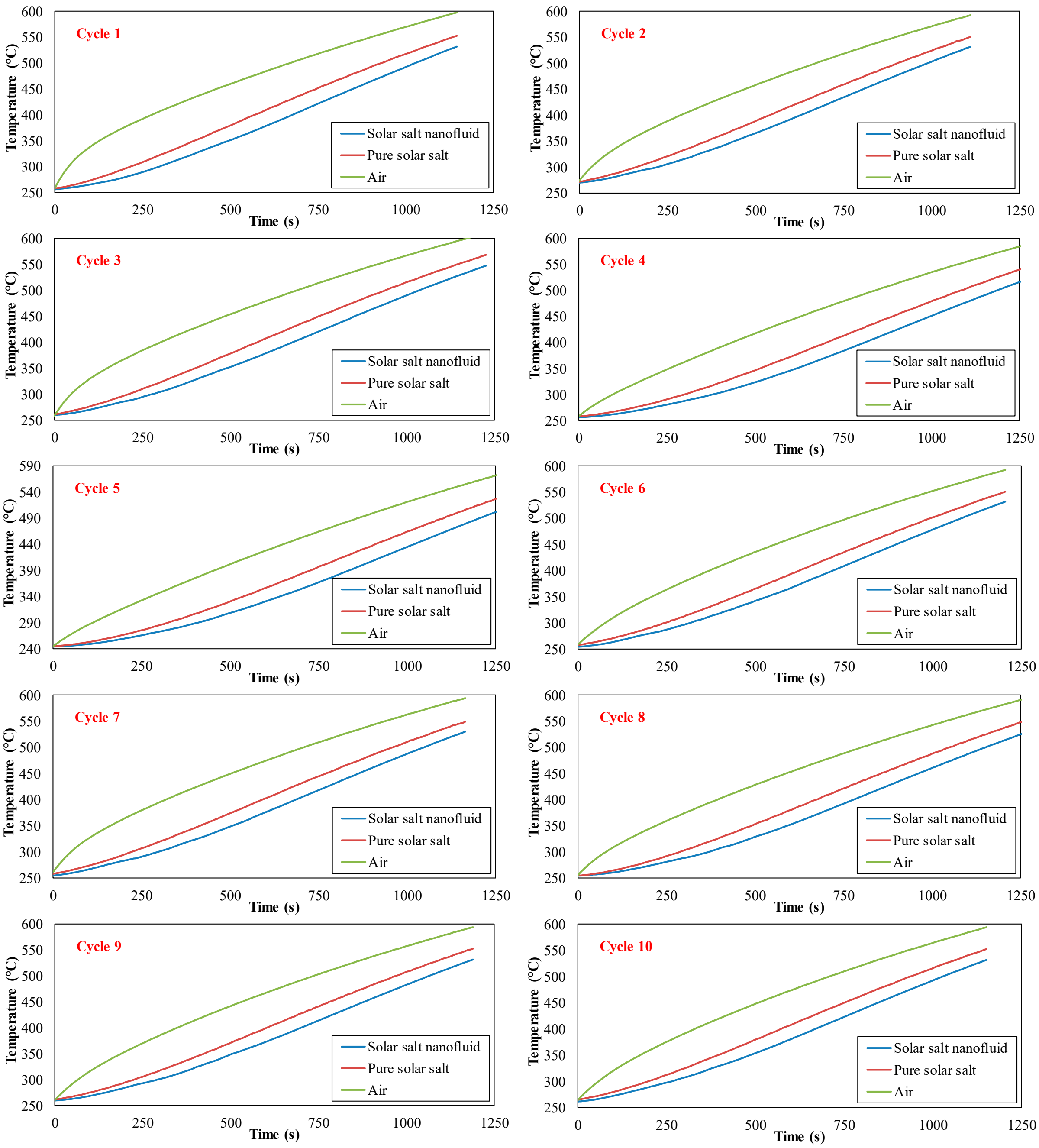

Figure S8. Plot of temperature response obtained from T-history experiments for thermocouples placed in air (in the furnace), pure solar salt samples and solar salt nanofluid samples (prepared using one-step synthesis protocol). The molten salt nanofluid samples were obtained by adding $Al(NO_3)_3{\cdot}9H_2O$ at 6.9% mass fraction (as nanoparticle precursors for yielding nanofluids) to pure solar salt for a target mass fraction of 1% for the resulting Al_2O_3 nanoparticles obtained by thermal decomposition (one-step synthesis protocol).

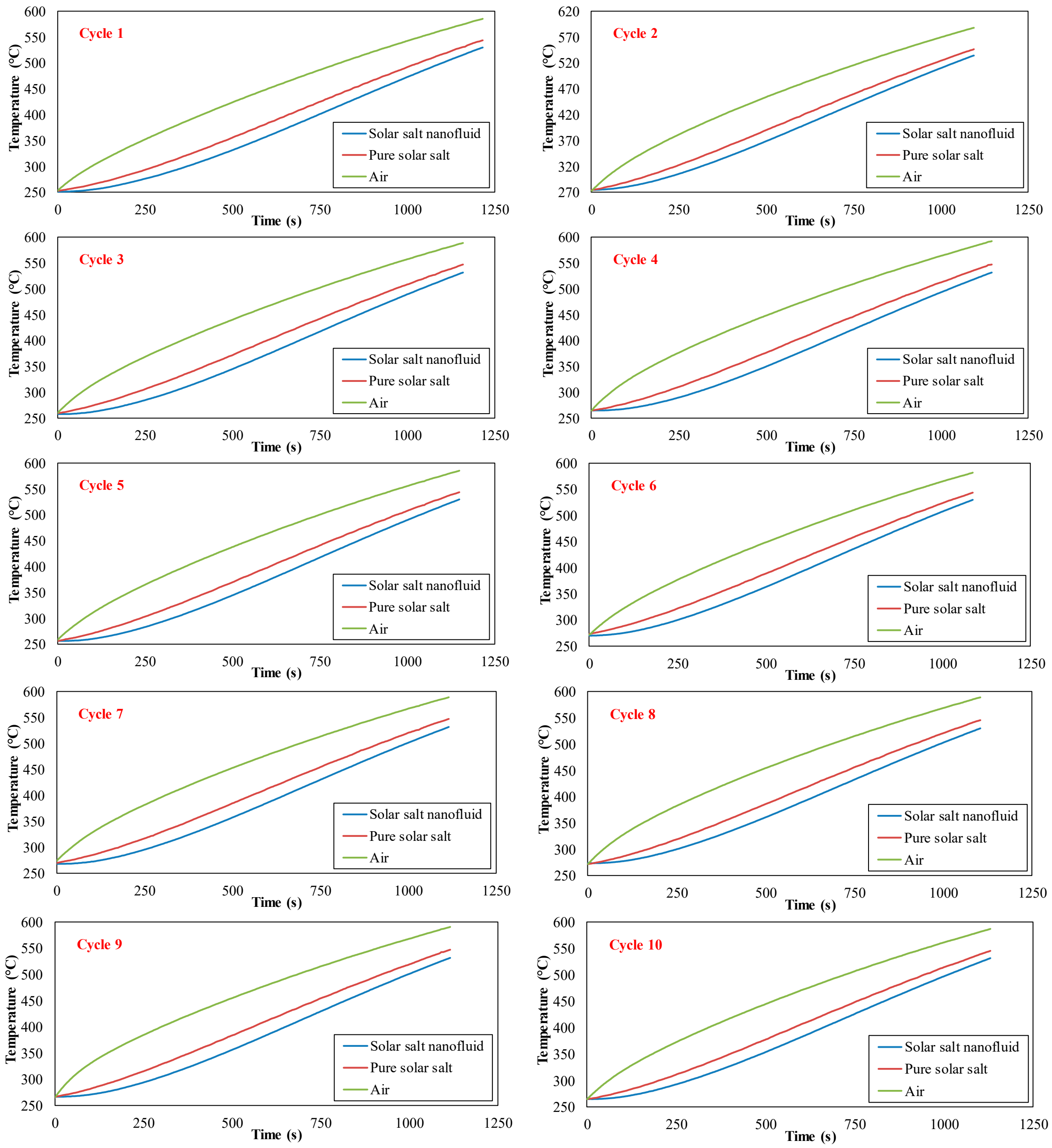

Figure S9. Plot of temperature response obtained from T-history experiments for thermocouples places in air (in the furnace), pure solar salt samples and solar salt nanofluid samples (prepared using one-step synthesis protocol). The molten salt nanofluid samples were obtained by adding $Al(NO_3)_3{\cdot}9H_2O$ at 10.1% mass fraction (as nanoparticle precursors for yielding nanofluids) to pure solar salt for a target mass fraction of 1.5% for the resulting Al_2O_3 nanoparticles obtained by thermal decomposition (one-step synthesis protocol).

S6. Validation of the Specific Heat Capacity Measurement of Solar Salt Nanofluid by Modulated Differential Scanning Calorimetry (MDSC)

Modulated differential scanning calorimetry (MDSC) experiments were performed to validate the results obtained from the T-history experiments for the specific heat capacity measurements of solar salt—1.0 wt.% alumina nanofluid sample. The results from the MDSC measurements are shown in Figure S10 below. The results from the MDSC experiments show that the average enhancement in the value of specific heat capacity measurements was ~32% when pure solar salt samples were mixed with additive [$Al(NO_3)_3{\cdot}9H_2O$] at 6.9% mass fraction for yielding Al_2O_3 nanoparticles at a target mass fraction of 1%. Such a result is in good agreement with the T-history test performed in this study where the level of enhancement is around 38% with an uncertainty of 7.5%.

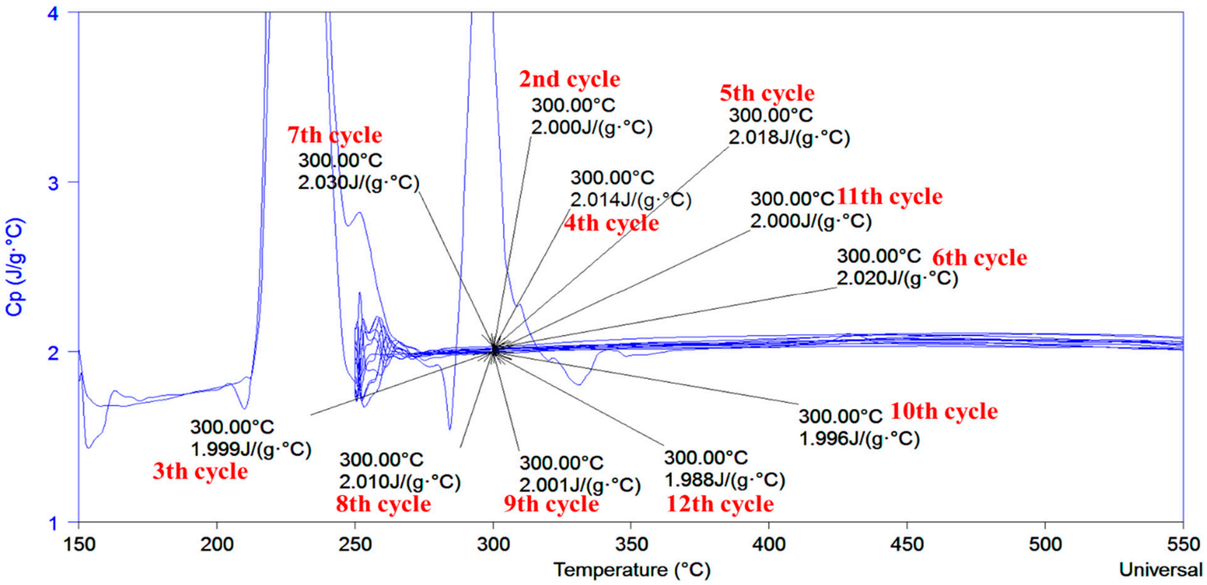

Figure S10. Plot of specific heat capacity as a function of temperature (obtained from MDSC experiments) for nanofluid samples containing Al_2O_3 nanoparticles at 1.0% mass fraction generated from $Al(NO_3)_3{\cdot}9H_2O$ precursor at 6.9% mass fraction.

S7. Energy-Dispersive X-ray Spectroscopy (EDS) Analysis of Nano-Clusters Observed in the Molten Salt Nanofluid Sample

As shown in Figure S11a, scattered nano-clusters (highlighted in red circle) are observed in the SEM images of the molten salt nanofluid containing alumina nanoparticles at 1.0% mass concentration. Pointwise energy-dispersive X-ray spectroscopy (EDS) analysis was performed on two different locations of the molten salt nanofluid sample at higher magnification, as shown in Figure S11b. As shown in the result, the elemental composition of these nanoparticle clusters is confirmed by the EDS spectrum which shows a lower level of Al on planar area but a significant elevation in the level of Al (which implies the presence of Al_2O_3 nanoparticles) in the nano-cluster region.

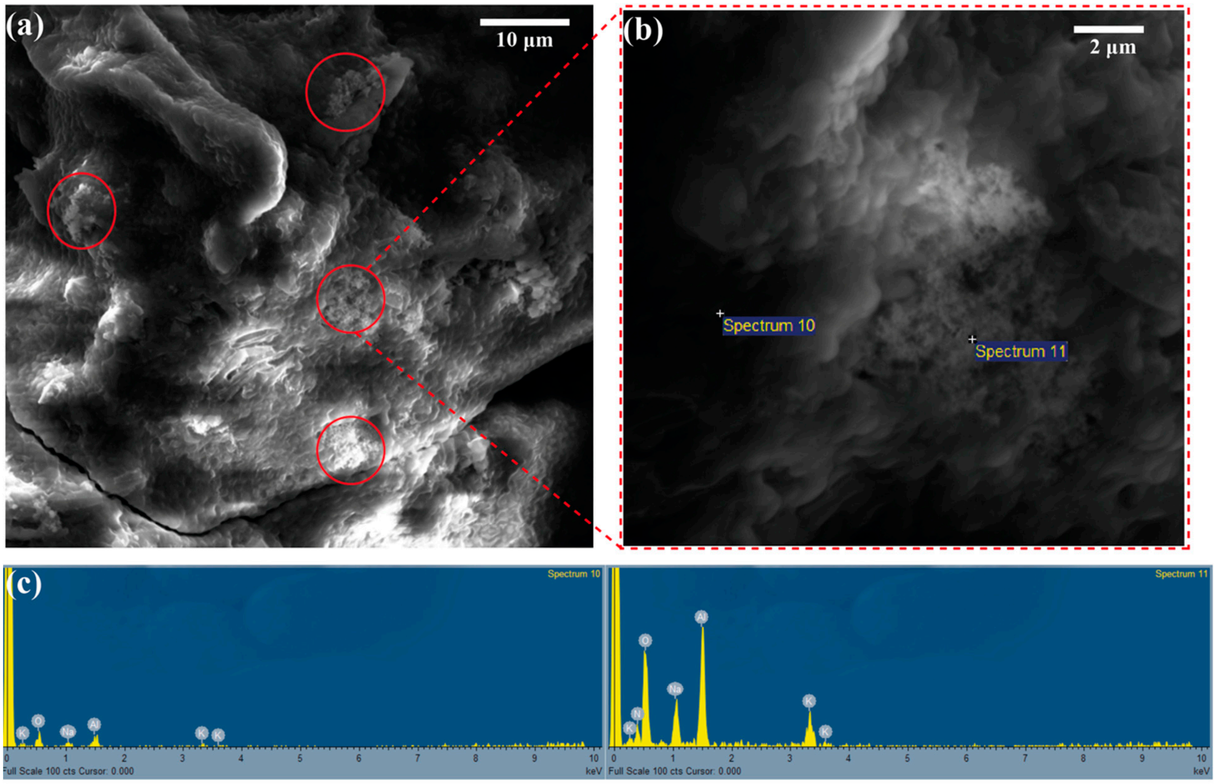

Figure S11. (**a**) Scanning electron microscopy (SEM) images of solar salt nanofluid samples with Al_2O_3 nanoparticles generated in-situ from the precursor under low magnification; (**b**) energy-dispersive X-ray spectroscopy (EDS) analysis of elemental composition of solar salt nanofluid at two different locations under high magnification; (**c**) the EDS spectra obtained at two different locations confirming the existence of aluminum element in the nano-cluster region.

S8. Size Analysis of Nanostructures Formed in the Solar Nanofluid Sample

Figure S12 shows the magnified scanning electron microscope (SEM) image taken at the cluster region in Figure S11b. The magnitude of the stem diameter was estimated at six different locations. While the limited magnification in SEM makes it challenging to obtain an exact average diameter or diameter distribution of the nano-cluster structure in the molten salt nanofluid sample, the result shown in the figure suggests that the size of the stem (i.e., length) of these nanostructures was around ~50 nm.

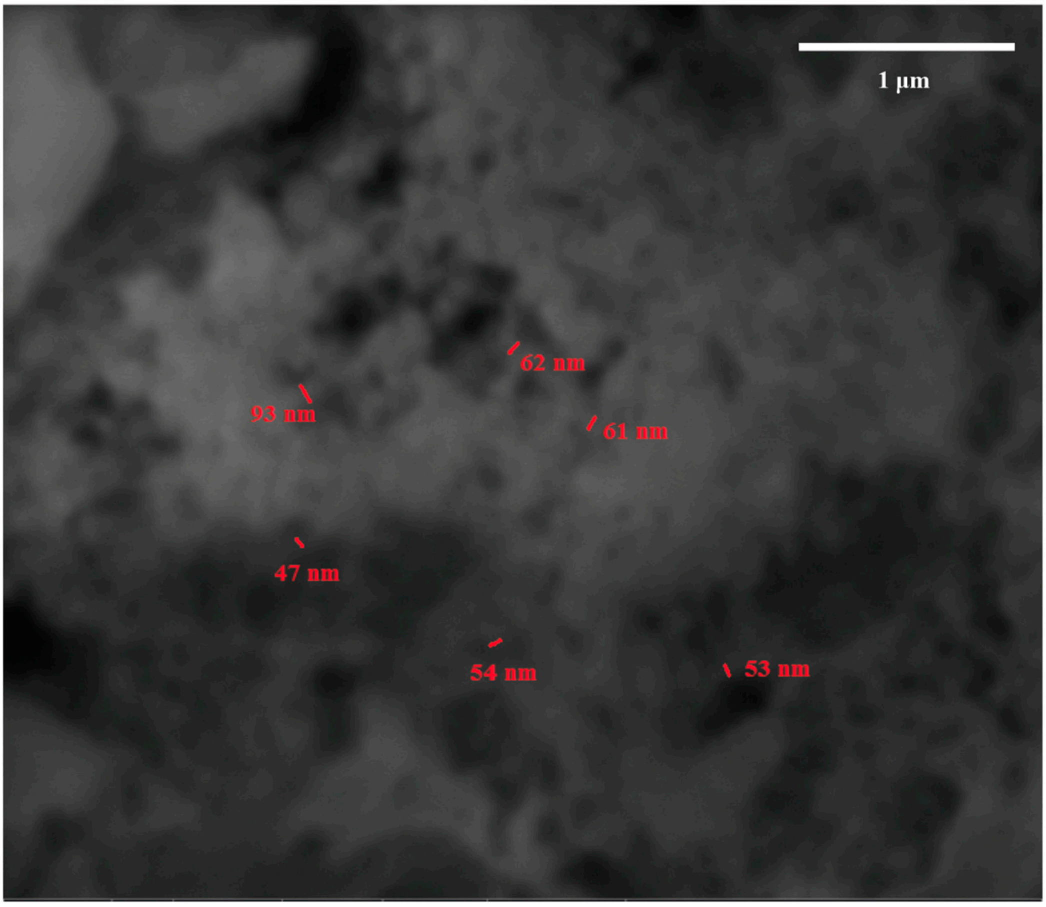

Figure S12. SEM image showing measurement of size of stem of nanostructures in the molten salt nanofluid sample containing Al_2O_3 nanoparticles synthesized in-situ from aluminum nitrate additives. The measurements show that the size of the stem of these nanostructures are around 50 nm.

S9. Estimating the Ratio between Kinetic Energy (KE) and Potential Energy (PE) for Compressed Layer in the Molten Nitrate System

The ratio between the kinetic energy and potential energy in the compressed phase molten nitrate salt system can be qualitatively estimated using a simple mathematical model based on one-dimensional oscillation of molecules, as shown in Figure S13. Figure S13 shows a one-dimensional representation of the bond interactions between one sodium ion and two collinear nitrate ions (in the form of spring-mass systems).

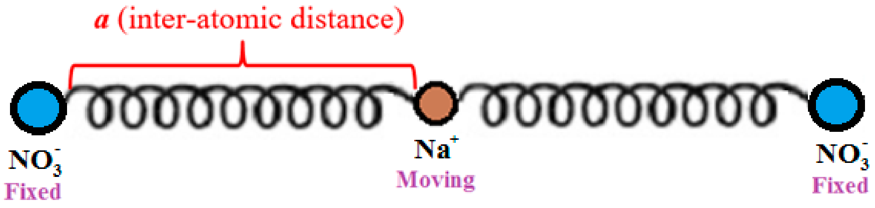

Figure S13. Schematic diagram of 1-D oscillation of sodium ion between two nitrate ions.

We assume the total potential energy associated with the moving atoms can be represented by Lennard–Jones potential (V_{LJ}) and electrostatic potential between a pair of charged particles as:

$$\mathrm{V}_{LJ} = 4\varepsilon\left[\left(\frac{\sigma}{r}\right)^{12} - \left(\frac{\sigma}{r}\right)^{6}\right] \tag{S1}$$

$$\mathrm{V}_{e} = \frac{ke^2}{r} \tag{S2}$$

where, ε is the depth of the potential well, σ is the finite distance at which the inter-particle potential is zero, r is the distance between the particles, k is the Coulomb's constant and e is the elementary charge of single electron. The inter-atomic force (F_{LJ}) can then be represented by taking the gradient of Equation S1 as:

$$\mathrm{F}_{LJ} = 4\varepsilon\left[6\left(\frac{\sigma}{r}\right)^{7} - 12\left(\frac{\sigma}{r}\right)^{13}\right] \tag{S3}$$

$$\mathrm{F}_{e} = \frac{ke^2}{r^2} \tag{S4}$$

The trajectory of the moving sodium ion can then be represented by the following equation:

$$\frac{\mathrm{d}^2x}{\mathrm{d}t^2} = \frac{4\varepsilon}{m}\left\{\left[6\left(\frac{\sigma}{a-x}\right)^{7} - 12\left(\frac{\sigma}{a-x}\right)^{13}\right] - \left[6\left(\frac{\sigma}{a+x}\right)^{7} - 12\left(\frac{\sigma}{a+x}\right)^{13}\right]\right\} + \frac{k}{m}\left[\frac{e^2}{(a-x)^2} - \frac{e^2}{(a+x)^2}\right] \tag{S5}$$

where, $\boldsymbol{x}$ = 0 is defined at the midpoint of two nitrate ions and $\boldsymbol{a}$ is the half distance between two nitrate ions. By perturbing the system with an initial velocity of the center atom at $\boldsymbol{t}$ = 0, the velocity and displacement profile of the particle with respect to time can be numerically evaluated using the classic Velocity Verlet algorithm, by converting the equation into a set of first order ordinary differential equations. The intermolecular parameters for sodium nitrate were obtained from the standard library of Material Studio (Accelrys, Inc., 2008) and are summarized in Table S3. Figure S14 shows the example particle trajectory and velocity profile calculated with a = 0.5 Å and a = 1.5 Å.

Table S3. Parameters for intermolecular interactions for sodium nitrate.[1]

Atom	q (e)	ε (kcal/mol)	σ (Å)
Na	+1	0.086	2.730
N	+0.95	0.080	3.900
O	-0.65	0.155	3.154

[1] The cross terms were computed using the mixing rule: $\varepsilon_{ij} = \left(\varepsilon_{ii}\varepsilon_{jj}\right)^{1/2}$, $\frac{1}{\sigma_{ij}} = \frac{1}{\sigma_{ii}} + \frac{1}{\sigma_{jj}}$

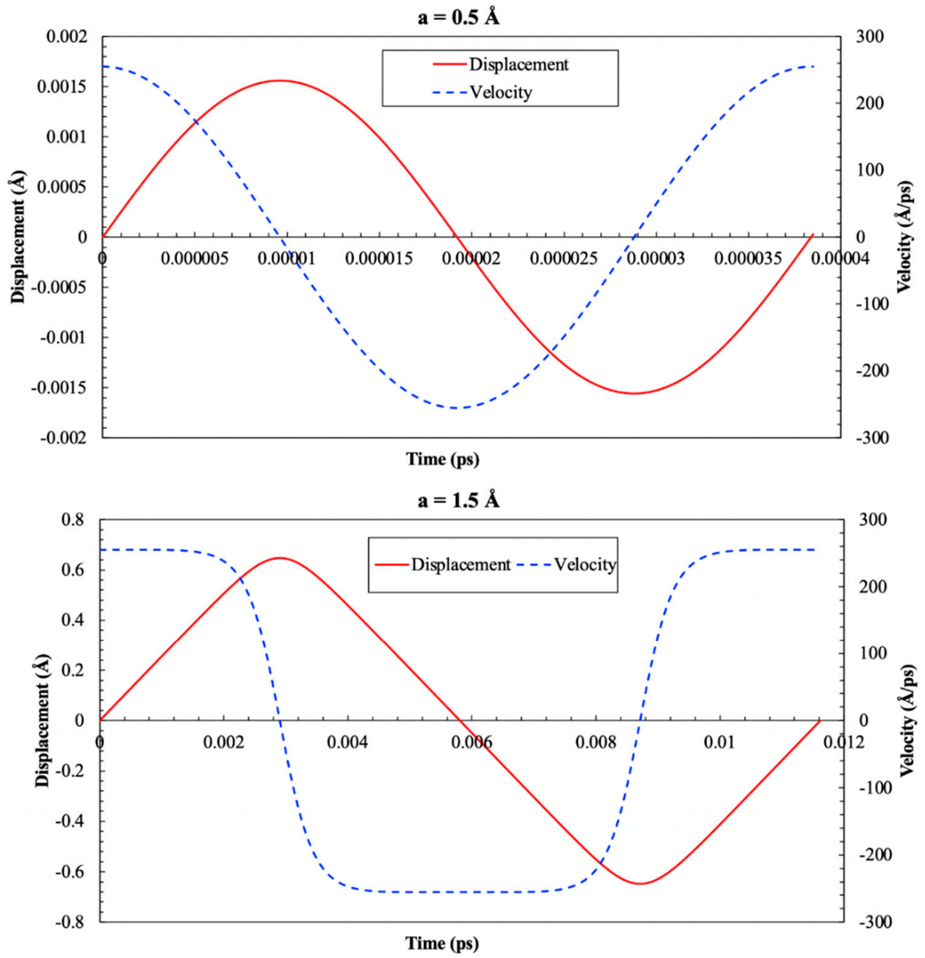

Figure S14. Displacement and velocity profile of oscillating sodium ion as a function of interatomic distance with nitrate ions: (**top**) a = 0.5 Å; (**bottom**) a = 1.5 Å.

On reducing the intermolecular distance ***a*** from 1.5 Å to 0.5 Å the time averaged values of KE and PE obtained from these equations for one complete cycle of oscillation, it was observed that the percentage of KE (in the total internal energy) reduces dramatically as the intermolecular spacing is decreased (as shown in Table S4 and Figure S15). The predictions from this model implies that the effective specific heat capacity of closely packed molecules is increased with increase in packing density, since additional quantities of energy is required (i.e., the total internal energy is higher) in order to maintain the system in thermal equilibrium or under isothermal conditions (i.e., for the same KE or for the same level of translational motion of the particles). Hence, this proves that the specific heat capacity of the compressed phase of the molten salt is significantly higher than that of the bulk phase.

Table S4. Estimation of kinematic energy (KE), potential energy (PE) and total internal energy (PE + KE) as a function of the intermolecular distance between sodium and nitrate ions. The calculated values were derived from Equation S5 and the parameters are listed in Table S3.

a (Å)	Energy (×10⁻¹⁸ J)				$\frac{KE}{PE}$	$\frac{KE}{KE+PE}$
	KE	PE (Lennard-Jones)	PE (electrostatic)	PE (total)		
0.5	6.307	16.373	9.231	25.604	0.246	20%
0.6	6.236	1.837	7.693	9.530	0.654	40%
0.7	6.256	0.293	6.595	6.888	0.908	48%
0.8	6.351	0.0631	5.777	5.840	1.087	52%
0.9	6.608	0.019	5.153	5.172	1.277	56%
1	7.182	0.009	4.673	4.682	1.533	61%
1.1	7.938	0.006	4.299	4.304	1.844	65%
1.2	8.668	0.004	3.997	4.001	2.166	68%
1.3	9.287	0.003	3.745	3.748	2.477	71%
1.5	10.165	0.003	3.343	3.346	3.038	75%

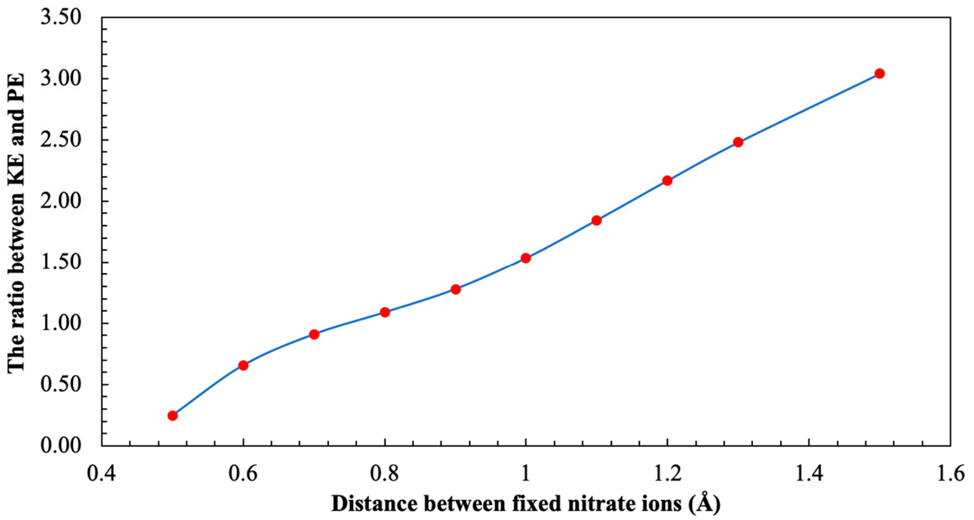

Figure S15. Plot for ratio of kinetic energy to potential energy as a function of intermolecular distance between sodium and nitrate ions. The plot was derived from Equation S5 and the parameters are listed in Table S3.

References

1. Incropera, F.P.; DeWitt, D.P.; Bergman, T.L.; Lavine, A.S. Section 3.6.4 Fins of Non-Uniform Cross-Sectional Area. In *Fundamentals of Heat and Mass Transfer*, Incropera, F.P., Ed.; John Wiley & Sons: Hoboken, NJ, USA, 2006.
2. Kawaizuni, F.; Otake, T.; Nomura, H.; Miyahara, Y. Heat capacities of aqueous solutions of ethylene glycol, propylene glycol and 1, 3-butanediol. *Nippon Kagaku. Kaishi* **1972**, *1972*, 1773–1776.
3. Katayama, T. Heats of mixing, liquid heat capacities and enthalpy-concentration charts for methanol–water and iso-propanol–water systems. *Kagaku Kogaku* **1962**, *26*, 361–372.
